# Pleiotropic Meta-Analysis of Age-Related Phenotypes Addressing Evolutionary Uncertainty in Their Molecular Mechanisms

**DOI:** 10.3389/fgene.2019.00433

**Published:** 2019-05-10

**Authors:** Alexander M. Kulminski, Yury Loika, Jian Huang, Konstantin G. Arbeev, Olivia Bagley, Svetlana Ukraintseva, Anatoliy I. Yashin, Irina Culminskaya

**Affiliations:** Biodemography of Aging Research Unit, Social Science Research Institute, Duke University, Durham, NC, United States

**Keywords:** genetic association studies, pleiotropy, age-related phenotypes, genetic heterogeneity, aging, health span, life span

## Abstract

Age-related phenotypes are characterized by genetic heterogeneity attributed to an uncertain role of evolution in establishing their molecular mechanisms. Here, we performed univariate and pleiotropic meta-analyses of 24 age-related phenotypes dealing with such evolutionary uncertainty and leveraging longitudinal information. Our analysis identified 237 novel single nucleotide polymorphisms (SNPs) in 199 loci with phenotype-specific (61 SNPs) and pleiotropic (176 SNPs) associations and replicated associations for 160 SNPs in 68 loci in a modest sample of 26,371 individuals from five longitudinal studies. Most pleiotropic associations (65.3%, 115 of 176 SNPs) were impacted by heterogeneity, with the natural-selection—free genetic heterogeneity as its inevitable component. This pleiotropic heterogeneity was dominated (93%, 107 of 115 SNPs) by antagonistic genetic heterogeneity, a phenomenon that is characterized by antagonistic directions of genetic effects for directly correlated phenotypes. Genetic association studies of age-related phenotypes addressing the evolutionary uncertainty in establishing their molecular mechanisms have power to substantially improve the efficiency of the analyses. A dominant form of heterogeneous pleiotropy, antagonistic genetic heterogeneity, provides unprecedented insight into the genetic origin of age-related phenotypes and side effects in medical care that is counter-intuitive in medical genetics but naturally expected when molecular mechanisms of age-related phenotypes are not due to direct evolutionary selection.

## Introduction

Large-scale genetic association studies including those called genome-wide association studies (GWAS) are a powerful tool for gaining insight into the genetics of human health span and life span. Historically, such studies were built in the framework of medical genetics, which is best adapted for Mendelian (hereditary) disorders. Major contributors to health span are common diseases (such as cardiovascular disease, stroke, cancer, Alzheimer's disease) occurring in late (i.e., post-reproductive) life and the risk factors that make individuals vulnerable to these diseases, collectively called age-related phenotypes. These are complex phenotypes of non-Mendelian type (Plomin et al., 2009). However, large-scale genetic association studies of such phenotypes utilize the same framework as for Mendelian disorders.

Biologists argue that gaining insight into biological processes requires evolutionary explanations (Dobzhansky, 1973). Because medical genetics historically focused on Mendelian-type hereditary disorders, such disorders are traditionally explained in terms of basic evolutionary forces such as mutation, natural selection, genetic drift, and gene flow. However, the sister discipline of evolutionary biology emphasizes fundamental difficulties in genetics of age-related phenotypes due to an uncertain role of evolution in establishing their molecular mechanisms (Kirkwood et al., 2011; Nesse et al., 2012). This problem is complicated by recent changes in human life expectancy (Oeppen and Vaupel, 2002) and the fitness landscape (Vijg and Suh, 2005; Crespi et al., 2010). Paradoxically, both medical genetics and evolutionary biology developed mostly independently, despite their reliance on the same forms of genomic data.

Evolutionary biology argues that mechanisms of age-related phenotypes are the results of *indirect* factors (“side-effects”) such as co-evolution with fast-evolving pathogens, mismatch with environments, reproductive success at the expense of health, trade-offs that leave every trait suboptimal, defenses and their special costs (Nesse and Williams, 1994; Nesse et al., 2012). The indirect role of evolution in mechanisms of age-related phenotypes, called in this paper evolutionary uncertainty, is the source of natural-selection–free genetic heterogeneity in predisposition to age-related phenotypes. This is an inevitable source of heterogeneity in the case of age-related phenotypes complementing the traditionally considered sources associated with complexity of phenotypes and their polygenicity. The inherent heterogeneity of age-related phenotypes can explain why the same allele can confer different, even antagonistic, risks to the same phenotype in different populations with the same ancestry (Day-Williams and Zeggini, 2011; Ukraintseva et al., 2016) and may result in complex forms of pleiotropy with seemingly related (Kulminski et al., 2016c, 2017) or unrelated (Goh et al., 2007; Barabasi et al., 2011; Kulminski et al., 2016a) phenotypes. This complexity implies that the role of the same genetic variant in the same phenotype can be naturally modified by the life course (Kulminski et al., 2013, 2017; Ukraintseva et al., 2016) regardless of heritability of such phenotypes because heritability concept for age-related phenotypes is notoriously problematic (Lewontin, 1974; Rose, 2006).

Here, we report the results of univariate and pleiotropic meta-analyses of genetic associations with multiple age-related phenotypes dealing with the evolutionary uncertainty in establishing their molecular mechanisms and leveraging longitudinal information. This approach follows the framework of evolutionary biology, which argues that genetic predisposition to age-related phenotypes is inherently heterogeneous, with the natural-selection–free genetic heterogeneity as its inevitable component. Thus, the key element of this biologically-motivated approach is addressing the inherent heterogeneity in genetic predisposition to such phenotypes. We identified 411 non-proxy single nucleotide polymorphisms (SNPs) (with linkage disequilibrium *r*^2^ < 70%) with genome-wide (GW) significance (*p* < *p*_*GW*_ = 5 × 10^−8^) in a modest sample of 26,371 Caucasians from five longitudinal studies, including 237 novel SNPs (199 loci), 11 SNPs in the major histocompatibility complex (MHC), and 3 (3 loci) and 160 (68 loci) SNPs with *p*-values smaller and larger, respectively, in the current study than in previous studies. We show that the evolutionary uncertainty plays a dominant role in the associations with age-related phenotypes. We found that vast majority of pleiotropic associations with age-related phenotypes were affected by antagonistic genetic heterogeneity, a phenomenon not previously routinely recognized, that is characterized by antagonistic directions of genetic effects for directly correlated phenotypes.

## Materials and Methods

### Study Cohorts

Data were obtained from five longitudinal studies from the Candidate gene Association Resource, Atherosclerosis Risk in Communities (ARIC) study (Investigators, 1989), Cardiovascular Health Study (CHS) (Fried et al., 1991), Coronary Artery Risk Development in Young Adults (CARDIA) study (Hughes et al., 1987), Multi-Ethnic Study of Atherosclerosis (MESA) (Bild et al., 2002), and Framingham Heart Study (FHS) (Cupples et al., 2009). The FHS included three cohorts comprising parental (FHS_C1), offspring (FHS_C2), and grandchildren (FHS_C3) generations. Given this complex design, the FHS cohorts were examined separately.

### Phenotypes

The analyses focused on 24 phenotypes (16 quantitative markers, 7 diseases, and death) listed in Table 1. Given the longitudinal design of the studied cohorts, the analyses leveraged repeated measurements of quantitative markers during follow-up and information about the timing of disease onset or death (Supplementary Table 1). These not strongly correlated phenotypes (Supplementary Figure 1) were available in the majority of selected cohorts. All studies collected information on diseases and death in population samples during follow-up.

**Table 1:** Basic characteristics of cohorts included in the analyses.

**Cohort**	**Sample size**	**Number of visits**	**Age (SD), years**	**Birth dates (range)**	Quantitative markers^*^	Risk outcomes^**^
ARIC	10,540	4	54.3 (5.7)	1920–1945	All except ADPN and AlbU	All except ND
CHS	4,174	10	72.8 (5.6)	1885–1925	All except IL6	All
FHS_C1	639	28	35.7 (4.3)	1895–1920	All except ADPN, AlbU, CRP, and IL6	All
FHS_C2	3,062	8	34.8 (9.8)	1910–1965	All	All
FHS_C3	3,960	2	40.2 (8.8)	1930–1980	All	Cancer only
MESA	2,474	5	62.7 (10.3)	1917–1957	All except HGB and AlbS	All except ND
CARDIA	1,522	6	25.6 (3.4)	1950–1965	All except ADPN and IL6	DM only

* *Quantitative markers are grouped in three domains: physiological (PHY): body mass index (BMI), diastolic blood pressure (DBP), forced expiratory volume in 1 second (FEV1), heart rate (HR), systolic blood pressure (SBP); blood (BLD): adiponectin (ADPN), albumin in serum (AlbS), blood glucose (BG), creatinine, hemoglobin (HGB), high-density lipoprotein cholesterol (HDL-C), total cholesterol (TC), triglycerides (TG); and inflammation (INF): albumin in urine (AlbU), C-reactive protein (CRP), interleukin 6 (IL6)*.

** *Risk outcomes: atrial fibrillation (AF), cancer, coronary heart disease (CHD), diabetes mellitus (DM), death, heart failure (HF), dementia of Alzheimer type (ND), stroke*.

*Cohort: ARIC, Atherosclerosis Risk in Communities Study; CHS, Cardiovascular Health Study; FHS, Framingham Heart Study original cohort (FHS_C1), FHS_C2, FHS 2nd generation cohort; FHS_C3, FHS 3rd generation cohort; MESA, Multi-Ethnic Study of Atherosclerosis; CARDIA, Coronary Artery Risk Development in Young Adults*.

*Age is given at baseline; standard deviation (SD)*.

*Additional details are provided in Supplementary Table 1*.

### Genotypes

Genotyping in each study was performed using the same customized Illumina CVDSNP55v1_A chip with approximately 50,000 SNPs from more than 2,000 selected candidate genes. SNPs were included in the analyses after quality control in each study (call rate > 95%, Hardy-Weinberg disequilibrium *p* > 10^−4^, minor allele frequency [MAF] > 2%). In case of marginally smaller MAF for prioritized SNPs in a specific cohort compared with the MAF cut-off, these SNPs were used regardless of the MAF cut-off.

### Genes and Loci

SNPs were mapped to genes using variant effect predictor from Ensembl and the NCBI SNP database (assembly GRCh38.p7). Because the customized Illumina CVDSNP55v1_A chip was enriched by genes, most SNPs identified in our analyses were within or near protein-coding genes. If an index SNP was not within a protein-coding gene, the closest gene was assigned. Multiple genes were assigned if they were at about the same distance up- and down-stream from the index SNP or if the index SNP was within the region of overlapping genes. Loci were naturally associated with genes.

**Figure 1:**
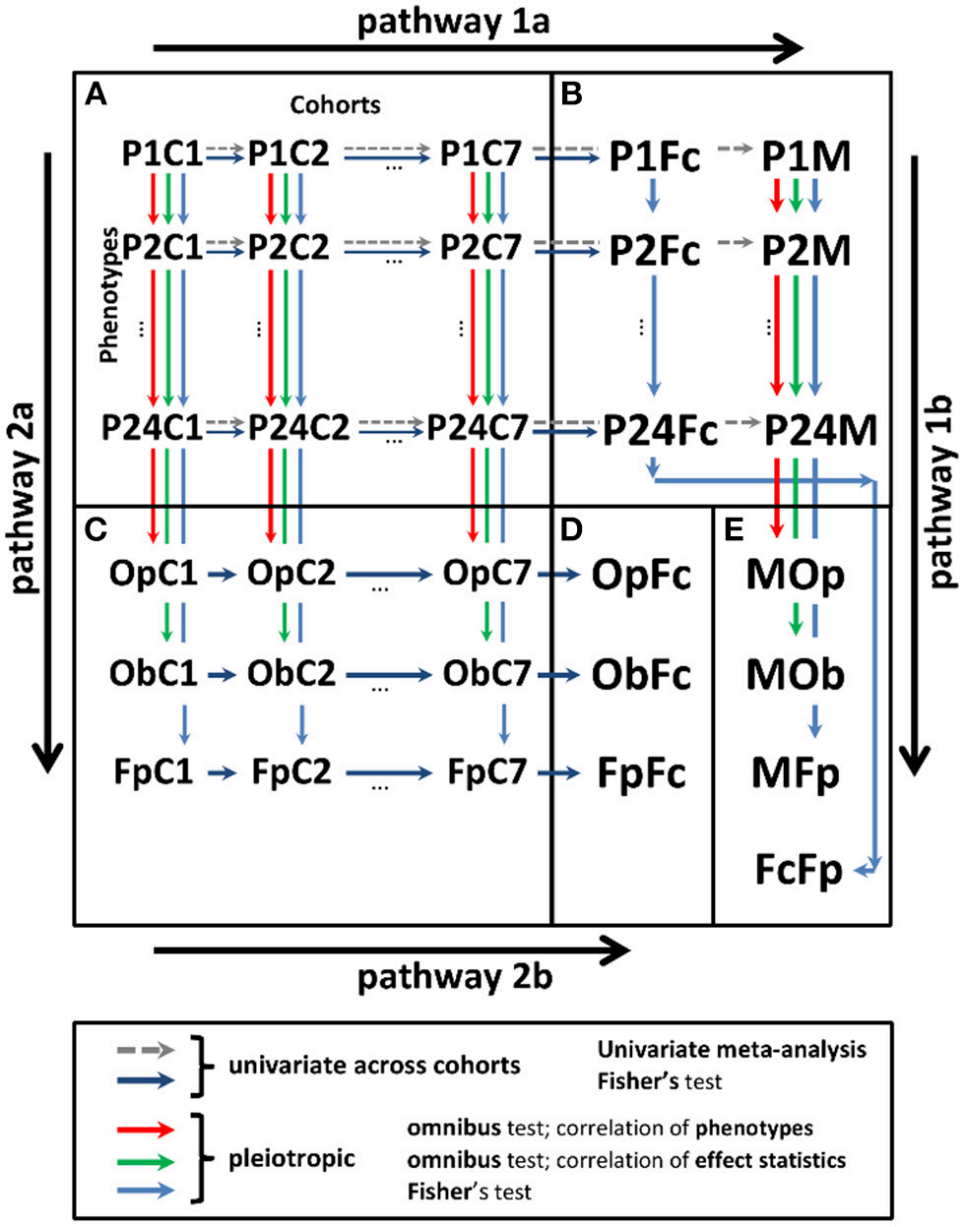
Scheme of univariate and pleiotropic meta-analyses in stage 2. **(A)** Statistics from stage 1 univariate genetic association study of 24 phenotypes in seven cohorts denoted P*i*C*j*, $i\in \left(\bar{1,24}\right)$ and $j\in \left(\bar{1,7}\right)$. **(B)** Univariate statistics from the meta-analysis across cohorts using the fixed-effects meta-test (P*i*M) and Fisher test (P*i*Fc). **(C)** Statistics from the pleiotropic meta-analysis across phenotypes in cohort *j* for: (i) omnibus tests with correlation matrix for phenotypes $\Sigma_{j}^{P}$ (OpC*j*) and effect statistics $\Sigma_{j}^{B}$ (ObC*j*) and (ii) Fisher test (FpC*j*). **(D)** Meta-statistics from Fisher test across cohorts for the results in **(C)** (OpFc, ObFc, and FpFc). **(E)** Meta-statistics across phenotypes for the results in **(B)** from (i) meta-test across cohorts and omnibus test across phenotypes with correlation matrix for phenotypes $\Sigma_{m}^{P}$ (MOp), (ii) meta-test across cohorts and omnibus test across phenotypes with correlation matrix for effect statistics $\Sigma_{m}^{B}$ (MOb), (iii) meta-test across cohorts and Fisher test across phenotypes (MFp), and (iv) Fisher test across cohorts and Fisher test across phenotypes (FcFp). Pathway 1: meta-analysis combining statistics across cohorts (pathway 1a) and pleiotropic meta-analysis across phenotypes (pathway 1b). Pathway 2: meta-analysis combining statistics across phenotypes (pathway 2a) and cohorts (pathway 2b).

Analyses were performed in two stages (Figure 1).

### Univariate Genetic Association Study in Stage 1

Genetic association study was performed for each phenotype in each cohort separately. This conventional approach was enhanced by leveraging longitudinal information. For quantitative markers, we used all measurements available during follow-up for the same individuals. Information on longitudinal measurements has multiple advantages including a potential gain in statistical power. To correct for repeated measurements correlations in the analyses of quantitative markers in all studies except the FHS, we used the generalized estimating equation (GEE) model with random effects for repeated measurements (*gee* package in R used with unstructured correlations). As the FHS included participants from large families, we used the linear mixed effects multilevel model (*lme4* package in R) with random effects to correct for familial structure and repeated measurements correlations because the GEE model was not efficient due to memory constraints, particularly for variables with a large number of observations. Given gamma-like frequency distributions of ADPN, AlbU, CRP, and IL6, a GEE model with a gamma function and log-link was used in all studies. We evaluated associations for SNPs using the available measurements of quantitative markers for individuals of a given age at each examination.

Longitudinal information on time to events was implemented in the Cox proportional hazards mixed effects model (*coxme* package in R) that addressed familial relatedness. In the FHS, we used both prospective and retrospective onsets. The use of retrospective onsets in a failure-type model is justified by Prentice and Breslow (Prentice and Breslow, 1978). These analyses provide estimates of effects in a given population. The time variable in these analyses was the age at onset of an event or at right censoring.

The models were adjusted for: (all studies) age, sex, and the first five principal components; field center (ARIC, CARDIA, CHS, MESA); genotyping stage (CHS); and whether DNA samples had been subject to whole-genome amplification (FHS). Because the GEE model for AlbU with log-link gamma function adjusted for five principal components did not converge for CHS, this adjustment was disregarded in this analysis.

Principal component analysis (PCA) was performed on genotyping data after quality control using the *smartpca* program from the EIGENSOFT package.

### Meta-Analyses in Stage 2

After stage 1, each SNP had a table with association statistics for up to 24 phenotypes in seven cohorts. These statistics were combined along two possible pathways (Figure 1): (i) across studies and then across phenotypes and (ii) across phenotypes and then across studies. These pathways can provide different results because of the inherent heterogeneity in genetic predisposition to age-related phenotypes (see the Significance). To address the impact such heterogeneity, we used four tests in pathway 1 and three tests in pathway 2 (Figure 1).

In pathway 1, univariate (phenotype-specific) meta-analysis combining stage 1 statistics across cohorts (pathway 1a) was performed using the Fisher test and conventional GWAS fixed-effects meta-test. Pleiotropic meta-analysis (pathway 1b) was performed by combining the univariate meta-statistics for the same SNPs across phenotypes. In pathway 1b, we used the Fisher test and two omnibus tests. The latter are traditionally used to address correlations between the effect statistics and phenotypes (details on all tests are below).

In pathway 2, we performed first pleiotropic meta-analysis by combining the stage 1 univariate statistics across phenotypes in each cohort separately (pathway 2a) using the Fisher test and the two omnibus tests as in pathway 1b. Then, we combined these pleiotropic meta-statistics across cohorts using the Fisher test (pathway 2b).

### Fixed-Effects Meta-Test

We adopted a conventional GWAS meta-test using a fixed effects model with inverse-variance weighting [METAL software (Willer et al., 2010)]. The combined effect size was estimated as $\hat{\beta_{M}}=\left(\underset{i}{\sum }w_{i}\hat{\beta_{i}}\right)/\left(\underset{i}{\sum }w_{i}\right)$, and the variance of this effect-size was $var(\hat{\beta_{M})}=1/\left(\sum_{i}w_{i}\right)$, where $\hat{\beta_{i}}$ is the effect size in the study *i* and *w*_*i*_ is the reciprocal of the variance of $\hat{\beta_{i}}$.

### Fisher Test

The Fisher test (Fisher, 1970) combines *p-*values assuming that there is no correlation between the tests that generated these *p-*values. In pathway 1a, this test has the power to reject the “null” hypothesis of no pooled effect regardless of the effect sizes and directions in the cohort-specific statistics. Accordingly, this test can indicate heterogeneity in genetic associations by providing smaller *p-*values than those from the fixed-effects meta-test. In pathways 1b and 2a, the Fisher test combines *p*-values across phenotypes assuming that these *p-*values are from independent associations. This test is often used for pleiotropic meta-analysis of modestly correlated phenotypes (Fortney et al., 2015). Because the Fisher test combines *p*-values from multiple statistics, it addresses the problem of multiple testing by increasing the number of degrees of freedom.

### Omnibus Tests

The Fisher test is based on the assumption of independence of the combined statistics. The statistics for the associations of the same SNPs with different phenotypes may or may not be independent. It is therefore argued that tests penalizing for correlation of such statistics should be used to deflate the Fisher test estimates. A commonly adopted test in this case is an omnibus test (Xu et al., 2003; Bolormaa et al., 2014; Zhu et al., 2015).

Suppose that for a certain SNP we have an estimated effect size ${\hat{\beta }}_{ij}$ and its standard error ${\hat{\sigma }}_{ij}$ for the phenotype $i\in \left({\bar{1,K}}_{j}\right)$ in the cohort $j\in \left(\bar{1,7}\right)$, where *K*_*j*_ is the number of phenotypes in a study *j*. A general omnibus test statistic can be constructed as ${{\hat{z}}^{\prime }}_{j}\Sigma_{j}^{-1}{\hat{z}}_{j}$, where ${\hat{z}}_{j}={\hat{\beta }}_{j}/{\hat{\sigma }}_{j}$ is a *z*-score vector of associations of SNPs with phenotypes and **Σ**_*j*_ is the correlation matrix of the *z*-scores to be estimated (Xu et al., 2003; Bolormaa et al., 2014). Accordingly, this test takes into account the correlation of the effect statistics for different phenotypes. Under the null hypothesis (*β*_***j***_** = 0**), the test statistic follows a chi-squared distribution with *K*_*j*_ degrees of freedom

$$\begin{array}{l} {{\hat{\text{z}}}^{\prime }}_{j}\Sigma_{j}^{-1}{\hat{\text{z}}}_{j}\sim {\text{X}}_{K_{j}}^{2}\;, \end{array}$$

from which we obtained a combined *p-*value *p*_*j*_ in the study *j*.

Correlation matrices **Σ** can be evaluated from the estimates of the effect statistics for each phenotype for independent SNPs; they are denoted **Σ ≡ Σ**^***B***^. It has been argued that **Σ** can be also constructed by evaluating correlations between phenotypes (Zhu et al., 2015); these matrices are denoted **Σ ≡ Σ**^***P***^. Correlation matrices were constructed for pathways 1 and 2 (Figure 1) separately. Cohort-specific matrices $\Sigma_{\text{j}}^{\text{B}}$ for pathway 2 were constructed by evaluating correlations of the effect statistics in each cohort using the results from the univariate genetic association study in stage 1 for each phenotype for independent SNPs. For pathway 1, matrix $\Sigma_{\text{m}}^{\text{B}}$ was constructed by evaluating correlations of the effect statistics from the fixed effect meta-test of all cohorts. The phenotype-based cohort-specific matrices for pathway 2, $\Sigma_{j}^{P}$, were constructed using phenotype measurements in each cohort separately by evaluating correlations between vectors defined by person-observations of the selected phenotypes. In the case of no overlapping measurements of quantitative traits, the closest measurements to a given examination were used. Matrix $\Sigma_{m}^{P}$ for pathway 1 was constructed using a fixed effect meta-test applied to the correlation statistics of phenotypes in each cohort, which was evaluated using averaged values for quantitative traits measured longitudinally at different visits.

### Genetic Heterogeneity

Evolutionary biology argues that genetic predisposition to age-related phenotypes is inherently heterogeneous due to the undefined role of evolution in establishing their molecular mechanisms. This implies that mechanisms driving genetic predisposition to age-related phenotypes and correlations between these phenotypes may have different origins.

**Figure 2:**
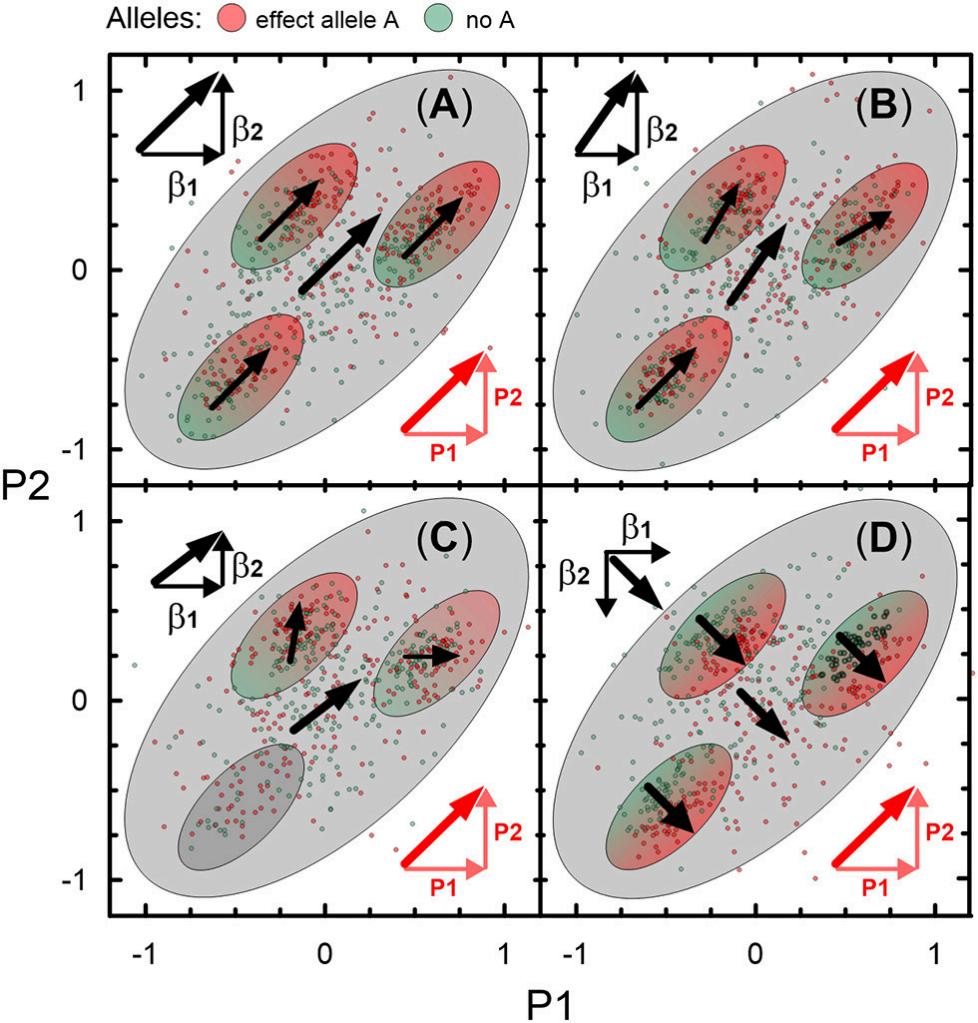
Schematic illustration of 4 types of genetic heterogeneity. Heterogeneity of: **(A)** type 1, **(B)** type 2, **(C)** type 3, and **(D)** type 4 in the associations with two partly correlated age-related phenotypes P1 and P2. Small dots represent a sample of carriers of an effect allele A (red color) and those who do not carry this allele (no A; green color). Large ellipse shows correlation of P1 and P2 in this sample (*r* = 0.6). Small ellipses show correlation of P1 and P2 in subsamples of this sample. Red color denotes vector of correlation of P1 and P2 (thick diagonal vector) and its projections on P1 (horizontal) and P2 (vertical). Black vectors β1 and β2 denote the effects in the associations of allele A with P1 and P2. Sum of β1 and β2 represents bivariate vector (thick line) of the effects.

Figure 2 illustrates four types of genetic heterogeneity in connections of SNPs with two partly correlated age-related phenotypes P1 and P2. Figures 2A,B illustrate commonly expected heterogeneities (of types 1 and 2) when vectors of the effects **β_1_** and **β_2_** are aligned with projections **P1** and **P2** of a vector of correlation, or, equivalently, a bivariate vector of the effects is aligned with a vector of correlation. Heterogeneity of type 1 (Figure 2A) refers to the situation when one observes perfect alignment of these vectors in some sub-samples. Although this situation resembles homogeneity, it actually may not be. To prove homogeneity, such perfect alignment should be in any sub-sample of a given sample. Type 2 (Figure 2B) is a commonly expected heterogeneity with a modest deviation from collinearity of the bivariate vector of the effects and the vector of correlation in subsamples and the entire sample. This heterogeneity, however, preserves alignment of these vectors in the same direction. Both types of heterogeneity can indicate shared mechanisms driving correlation between phenotypes and the effect statistics. Thus, either the **Σ**^***B***^ or **Σ**^***P***^-based omnibus test can penalize for this correlation, deflating the estimates from the Fisher test. This is evident from the test statistics ${{\hat{z}}^{\prime }}_{j}\Sigma_{j}^{-1}{\hat{z}}_{j}$ in the two-dimensional case,

(1) $$\begin{array}{r} {{\hat{z}}^{\prime }}_{j}\Sigma_{j}^{-1}{\hat{z}}_{j}=\left[\left({ẑ}_{1}^{2}\Sigma_{22}-{{ẑ}_{1}{ẑ}_{2}\Sigma }_{21}\right)+\left({ẑ}_{2}^{2}\Sigma_{11}-{{ẑ}_{1}{ẑ}_{2}\Sigma }_{12}\right)\right]/\text{det}\left(\Sigma \right) \end{array}$$

because ẑ_1_ẑ_2_Σ21, _ẑ_1_ẑ_2_Σ12_ > 0 in this situation. Penalization of these statistics indicates overlap in the association signals, which is essential for identifying mediated pleiotropy (Solovieff et al., 2013) and, potentially, shared biological mechanisms in genetic predisposition to different phenotypes.

Figure 2C illustrates less commonly expected heterogeneity of type 3 by showing that despite virtually the same alignment as in Figure 2B of the bivariate vector of the effects with the vector of correlation in the entire sample, the mechanisms are different. The difference is that the bivariate vector in Figure 2C is a superposition of virtually independent (orthogonal) bivariate vectors of the effects in subsamples. The subsample-specific bivariate vectors can be strongly misaligned with the vector of the phenotype correlation. Then, the omnibus **Σ**^***P***^-based test will be overly deflated compared to the Fisher and **Σ**^***B***^-based omnibus tests. Type 3 heterogeneity indicates biological pleiotropy due to mechanisms other than those driving correlation between the studied phenotypes.

Figure 2D illustrates heterogeneity of type 4, which is counter-intuitive in medical genetics but naturally expected when molecular mechanisms of age-related phenotypes are not due to direct evolutionary selection. This heterogeneity implies (virtual) orthogonality of the bivariate vector of effects to the vector of the phenotype correlation, which is driven by opposite directions of the effect vector (**β_2_** in this case) for one phenotype and projection of the vector of correlation on this phenotype (**P2**). Accordingly, this heterogeneity is called antagonistic heterogeneity, implying antagonistic directions of genetic effects for directly correlated phenotypes. In this case, the omnibus test provides smaller *p-*values than the Fisher test, as can be seen from (1) because ẑ_1_ẑ_2_Σ21, ẑ_1_ẑ_2_Σ12 < 0 in this case.

Genetic heterogeneity can be further complicated by subsamples with no effects (ellipse with no arrows in Figure 2C) and/or antagonistic effects for the same phenotypes at, e.g., different ages. This complication can make effects in the entire sample seemingly weak despite strong effects in subsamples.

### Significance and Novelty

The fixed-effect, Fisher, and two omnibus meta-tests used in our analyses have power to identify associations that are adjusted or unadjusted for correlation and heterogeneity in genetic predisposition to age-related phenotypes. Accordingly, GW level (*p* = 5 × 10^−8^) attained in either of these tests was used as a cut-off for significance for a given SNP. The difference between *p*-values from these tests for a SNP was used to characterize the impact of correlation and heterogeneity on the association.

SNPs were considered novel if they attained GW significance in: (i) the univariate meta-analysis but were not reported in the GRASP catalog (Leslie et al., 2014) at *p* ≤ 5 × 10^−8^ or (ii) the pleiotropic meta-analysis but not in the pleiotropic analysis of results from GRASP. For univariate meta-analysis, we used evidence for the same (index) SNP in our study and GRASP. If no associations were reported in GRASP for the index SNP, we included SNPs with the smallest *p*-values within ± 1 Mb flanking region. For pleiotropic meta-analysis, we selected associations from GRASP for the index SNPs with 24 phenotypes used in our analysis (Table 1). Then we performed pleiotropic meta-analysis by applying the Fisher test to these GRASP results to present evidence for pleiotropy in prior studies. We used the Fisher test because the effect sizes were not reported in GRASP. We adopted a conservative approach by not penalizing the Fisher statistics for phenotypes with *p-*values not reported in GRASP.

### Heterogeneity Coefficient

We used METAL software (Willer et al., 2010) to evaluate the heterogeneity coefficient I^2^. The I^2^ can be interpreted as the percentage of the total variability in a set of effect sizes due to between-sample variability.

## Results

### Study Overview

We used 24 weakly to moderately correlated (Supplementary Figure 1) age-related phenotypes (16 markers, seven diseases, and death) available from five longitudinal studies comprising seven cohorts (Table 1). Analyses were performed for 26,371 individuals of Caucasian ancestry, men, and women combined, in two stages using an additive genetic model with minor allele as an effect allele. In stage 1, we performed a genetic association study of each phenotype (one SNP—one phenotype) in each cohort separately (Figure 1A), following the traditional univariate GWAS design. This analysis leveraged longitudinal information from repeated measurements of quantitative markers and the timing of risk outcomes (Table 1 and Supplementary Table 1). The univariate statistics in each cohort for each phenotype (Figure 1A) can be combined via two pathways (Figures 1B–E). The first is the traditional pathway (pathway 1) meta-analyzing the results first across cohorts [as in most GWAS meta-analyses, e.g., (Willer et al., 2013)] and then across phenotypes (Visscher and Yang, 2016). The second pathway leverages the availability of the results from individual studies and, thus, it extends the methods based on the summary statistics (Visscher and Yang, 2016). Accordingly, for pathway 2, we first performed pleiotropic meta-analysis of the results across phenotypes in each cohort and then across cohorts. These analyses addressed the evolutionary uncertainty in establishing molecular mechanisms of age-related phenotypes by performing five meta-tests (Figure 1) (see “Materials and Methods”).

### Univariate Meta-Analysis and Heterogeneity

Using a Fisher test and conventional GWAS fixed-effect meta-test (Figure 1, pathway 1a), we identified 61 novel SNPs (see “Materials and Methods”) and 3 SNPs with smaller *p-*values than previously reported (55 loci; counts of loci exclude the MHC) (Table 2 and Supplementary Table 2). One SNP (rs10160664) was associated with two phenotypes, albumin in urine (AlbU) and C-reactive protein (CRP), at the GW level. Of these 65 GW significant associations (for 64 SNPs), 23 were identified in the fixed-effects meta-test, 11 in the Fisher test, and 31 in both tests. The Fisher test outperformed the meta-test (*p*_*Fisher*_ < *p*_*meta*_) for 18 of the 61 novel SNPs (29.5%). All associations were observed for quantitative markers: 52 for AlbU, 6 for total cholesterol (TC), 2 for heart rate (HR), 1 for high-density lipoprotein cholesterol (HDL-C), 1 for interleukin-6 (IL-6), 1 for albumin in serum (AlbS), 1 for adiponectin (ADPN), and 1 for CRP and AlbU. For 3 SNPs, GW significance in the current study was approximately the same as that in previous studies for the index SNPs (rs17645031, rs1784042) or a nearby SNP (rs1879266). The remaining 61 SNPs, or SNPs nearby to them, did not attain *p*_*GW*_ in previous studies (Table 2).

**Table 2:** Novel SNPs, and SNPs with smaller *p*-values than previously reported, attained genome-wide significance (*p* < 5 × 10^−8^) in univariate meta-analysis of individual phenotypes.

**ID**	**Locus^a^**	**SNP^b^**	**Chr**	**Location**	**EA**	**EAF**	**Trait**	**β_meta_**	**P_**meta**_**	**P_**Fisher**_**	**Ratio**	**I^2^ %**	**SNP${\text{SNP}}_{\text{known}}^{\text{c}}$**	**P_known_**	**LD %**
1	*TNFRSF1B*	rs5745938	1	12,165,317	a	0.043	AlbU	−0.44	4.78E-09	2.05E-09	0.96	78	rs5746040	9.40E-03	0.2
2	*PLA2G5*	rs11573294	1	20,091,603	g	0.023	AlbU	−0.66	8.75E-17	5.01E-16	1.05	67	rs2236772	4.00E-02	0.2
3	*TGFBR3*	rs17131556	1	91,768,996	g	0.042	AlbU	−0.40	2.14E-08	4.11E-08	1.04	65	rs2296621	7.30E-03	1.1
4	*MYBPHL*	rs17645031	1	109,292,316	a	0.083	TC	−4.87	6.01E-23	4.51E-19	1.21	0	rs17645031	1.12E-22	S
5	*F5*	rs9332664	1	169,517,708	t	0.026	AlbU	−0.51	3.40E-09	5.75E-11	0.83	82	rs6011	1.90E-03	0.1
6	*F5*	rs9332684	1	169,582,297	g	0.026	AlbU	−0.45	1.85E-08	1.14E-06	1.30	0	rs6011	1.90E-03	0.0
7	*RYR2*	rs10157718	1	237,093,677	t	0.023	AlbU	−0.48	3.55E-08	2.20E-11	0.70	86	rs12041754	1.10E-06	
8	*ABCG5/DYNC2LI1*	rs17031666	2	43,815,975	c	0.031	AlbU	−0.46	3.69E-09	3.64E-08	1.13	61	rs17031742	2.00E-02	0.5
9	*EPAS1*	rs17035091	2	46,383,270	g	0.037	AlbU	−0.49	1.24E-10	3.47E-09	1.17	47	rs7569138	6.30E-03	59
10	*MEIS1*	rs7563565	2	66,489,460	a	0.031	AlbU	−0.49	8.85E-10	2.52E-08	1.19	29	rs1055386	1.20E-04	1.5
11	*FABP1*	rs2970900	2	88,124,102	g	0.023	AlbU	−0.46	1.15E-08	1.15E-08	1.00	69	rs1545223	1.80E-02	0.1
12	*DPP4*	rs10490422	2	162,059,903	a	0.022	AlbU	−0.49	5.80E-08	8.13E-09	0.89	78	rs2892827	7.70E-03	0.8
13	*CPS1*	rs7558276	2	210,634,680	a	0.027	AlbU	−0.51	8.27E-10	6.22E-09	1.11	65	rs2371010	5.30E-04	0.0
14	*VHL*	rs17610448	3	10,153,488	c	0.024	AlbU	−0.49	2.66E-09	2.22E-07	1.29	4	rs9866514	3.30E-03	
15	*TGFBR2*	rs3087463	3	30,605,604	a	0.023	AlbU	−0.52	3.21E-10	9.51E-09	1.18	39	rs17026647	2.20E-06	
16	*FOXP1*	rs17747268	3	71,006,431	c	0.029	AlbU	−0.52	8.86E-12	3.87E-10	1.17	25	rs6549390	2.10E-03	0.8
17	*SLC34A2*	rs11731126	4	25,671,935	a	0.030	AlbU	−0.41	1.69E-07	3.67E-08	0.91	77	rs16876970	2.70E-04	
18	*TBC1D1*	rs4832741	4	37,895,064	g	0.022	AlbU	−0.53	1.75E-11	2.42E-10	1.12	55	rs1435385	4.60E-04	1.1
19	*FABP2*	rs1511024	4	119,319,026	a	0.033	AlbU	−0.44	9.81E-08	1.32E-08	0.89	74	rs17050049	1.00E-03	
20	SLC1A3^*^	rs2562544	5	36,524,655	g	0.021	AlbU	−0.56	1.42E-12	1.96E-11	1.11	56	rs298973	3.20E-03	0.8
21	*ADGRV1*	rs12522091	5	90,956,871	a	0.028	AlbU	−0.71	5.33E-13	1.18E-17	0.73	91	rs2935535	3.60E-04	2.8
22	GLRX^*^	rs17085170	5	95,836,627	a	0.051	AlbU	−0.40	2.78E-08	8.31E-07	1.24	32	rs10515242	3.60E-04	2.6
23	*F13A1*	rs3024471	6	6,319,302	g	0.028	AlbU	−0.48	2.17E-09	1.07E-08	1.09	56	rs4582427	9.90E-05	
24	*NFKBIL1*	rs3130062	6	31,558,135	g	0.069	TC	−3.32	1.53E-09	6.17E-08	1.22	46	rs3130062	1.60E-02	S
25	*MSH5*	rs3131382	6	31,739,953	a	0.069	TC	−3.44	2.47E-10	6.61E-09	1.17	53	rs3131382	9.24E-04	S
26	*RNF5/AGPAT1*	rs3134943	6	32,179,984	a	0.137	TC	−2.21	2.12E-08	1.53E-07	1.13	58	rs3134943	1.16E-04	S
27	HLA-DRA^*^	rs3129868	6	32,436,600	a	0.141	TC	−2.30	5.08E-09	9.58E-09	1.03	67	rs3129868	7.27E-04	S
28	*ESR1*	rs17761320	6	151,830,830	a	0.043	AlbU	−0.46	3.75E-08	7.08E-08	1.04	62	rs17761320	3.00E-02	S
29	*ESR1*	rs6913408	6	152,056,977	a	0.029	AlbU	−0.33	1.37E-04	4.14E-10	0.41	90	rs17081526	7.10E-04	0.0
30	*EGFR*	rs6968014	7	55,179,749	t	0.026	AlbU	−0.35	5.19E-05	2.86E-08	0.57	86	rs4495378	5.30E-04	1.2
31	*MLXIPL*	rs17145750	7	73,612,048	a	0.153	HDLC	0.947	2.49E-08	2.21E-05	1.63	0	rs17145750	6.05E-08	S
32	*HGF*	rs5745660	7	81,745,771	a	0.032	AlbU	−0.44	4.41E-09	7.29E-08	1.17	50	rs5745660	2.20E-02	S
33	*CDK5*	rs2069454	7	151,055,895	g	0.048	AlbU	−0.36	1.22E-08	5.71E-07	1.27	46	rs1860742	8.40E-06	
34	DEFA1^*^	rs12675298	8	6,971,291	a	0.023	AlbU	−0.42	1.12E-08	1.99E-08	1.03	70	rs7388463	2.70E-04	0.1
35	*MMP16*	rs11996222	8	88,204,251	a	0.061	AlbU	−0.38	1.22E-08	5.28E-07	1.26	0	rs7822721	4.40E-03	
36	*SQLE*	rs16900175	8	125,011,901	c	0.022	AlbU	−0.56	3.02E-11	4.35E-10	1.12	40	rs16900770	2.70E-05	
37	*GPR20*	rs36092215	8	141,357,146	a	0.040	AlbU	−0.46	2.94E-08	2.80E-06	1.36	0	rs10088001	2.30E-05	
38	*VLDLR*	rs35845312	9	2,620,592	a	0.022	AlbU	−0.48	4.54E-08	3.01E-08	0.98	72	rs7856406	1.20E-03	0.8
39	*CDKN2A*	rs3731249	9	21,970,917	a	0.030	AlbU	−0.48	1.83E-10	8.67E-10	1.07	69	rs7024096	2.70E-03	2.2
40	*ZFAND5*	rs13292980	9	72,358,555	a	0.066	AlbU	−0.43	1.96E-10	1.16E-08	1.22	22	rs11788021	1.40E-04	1.0
41	*SORCS1*	rs822008	10	106,784,843	g	0.024	IL6	7.73	1.70E-04	3.49E-09	0.45	91			
42	LRRC4C^**^	rs10160664	11	41,894,276	a	0.022	CRP	−0.18	1.40E-05	4.30E-09	0.58	86			
							AlbU	−0.50	4.25E-09	3.07E-07	1.29	0	rs10837551	4.20E-03	1.4
43	GIF/TCN1^*^	rs17597065	11	59,848,727	g	0.041	AlbU	−0.46	1.22E-07	2.80E-08	0.92	74	rs11230503	9.1E-05	0.0
44	*SIDT2*	rs1784042	11	117,194,760	a	0.402	TC	−1.54	2.49E-08	5.35E-06	1.44	0	rs1784042	3.83E-08	S
45	*CACNA1C*	rs2238017	12	2,069,379	g	0.329	HR	−0.47	1.92E-08	5.73E-06	1.47	0	rs2238017	3.37E-05	S
46	*CACNA1C*	rs2238018	12	2,069,495	a	0.187	HR	−0.62	5.94E-10	8.03E-08	1.30	19	rs2238017	3.37E-05	47
47	*BCAT1*	rs4963823	12	24,929,329	a	0.022	AlbU	−0.50	1.10E-06	2.31E-08	0.78	82	rs6487431	6.90E-04	
48	*PRKAG1*	rs1126930	12	49,005,349	c	0.032	AlbU	−0.44	1.73E-09	4.14E-08	1.19	29	rs11168354	3.50E-04	0.2
49	*LRP1*	rs1800137	12	57,154,683	a	0.022	AlbU	−0.59	2.69E-09	1.67E-08	1.10	51	rs11172106	1.8E-03	
50	*LRP1*	rs34577247	12	57,184,890	a	0.021	AlbU	−0.55	1.36E-10	1.78E-08	1.27	0	rs11172106	1.8E-03	0.5
51	*NTS*	rs11117072	12	85,882,569	a	0.034	AlbU	−0.46	9.23E-10	1.34E-07	1.31	3	rs10863085	2.00E-04	7.0
52	*DIABLO*	rs7957117	12	122,209,055	c	0.021	ADPN	−0.16	7.72E-09	8.72E-08	1.15	39	rs7957117	5.05E-05	S
53	*BNIP2*	rs1057058	15	59,672,705	a	0.035	AlbU	−0.41	1.08E-08	1.19E-06	1.34	0	rs8032049	6.30E-05	
54	AGRP^*^	rs13334182	16	67,490,121	a	0.022	AlbU	−0.44	1.69E-08	3.04E-08	1.03	66	rs8045523	2.70E-04	0.3
55	*POLR2A*	rs4265880	17	7,492,948	a	0.036	AlbU	−0.43	3.78E-08	5.22E-08	1.02	61	rs34780532	1.10E-03	
56	*GAS7*	rs9894339	17	10,049,295	g	0.024	AlbU	−0.56	5.14E-09	4.24E-09	0.99	74	rs16959324	2.40E-04	0.1
57	EIF1/GAST^*^	rs12949732	17	41,701,367	g	0.024	AlbU	−0.41	8.96E-06	4.85E-11	0.49	90	rs730086	6.30E-04	3.7
58	YES1^*^	rs9945303	18	813,556	g	0.033	AlbU	−0.52	2.25E-09	1.98E-12	0.74	87	rs2846758	2.00E-03	0.8
59	*BCL2*	rs4941192	18	63,276,726	g	0.040	AlbU	−0.43	4.00E-08	7.93E-07	1.21	35	rs7238248	1.70E-03	0.4
60	*BCL2*	rs17070959	18	63,283,686	a	0.034	AlbU	−0.52	1.32E-09	5.84E-08	1.23	25	rs7238248	1.70E-03	0.2
61	*NOTCH3*	rs12082	19	15,159,825	a	0.029	AlbU	−0.41	3.45E-08	3.01E-06	1.35	0	rs7250903	1.10E-04	0.1
62	*BAX*	rs4645886	19	48,956,428	a	0.023	AlbU	−0.53	5.96E-11	1.91E-09	1.17	32	rs10401192	5.50E-05	
63	*FLT3LG/RPL13A*	rs1879266	19	49,486,803	g	0.166	AlbS	−0.02	1.78E-08	4.11E-06	1.44	0	rs739347	3.20E-08	66
64	ERVV-1*	rs1650940	19	53,004,046	g	0.083	AlbU	−0.42	2.08E-07	1.39E-08	0.85	78	rs6509825	2.80E-04	0.5

a *Because the customized Illumina CVDSNP55v1_A array was gene enriched, loci were naturally associated with genes. If an index SNP was not within protein coding gene, the closest gene(s) were assigned. Multiple genes were selected if they were at about the same distance up- and downstream from the index SNP or the index SNP was within the region of overlapping genes*.

* *genes within ± 100 Kb flanking region for the index SNP*.

** *LRRC4C gene laid 435 Kb apart of rs10160664*.

b *Proxy SNPs with linkage disequilibrium (LD) r^2^ > 70% were excluded. Four SNPs on chromosome 6 (24–27) and two SNP pairs on chromosomes 12 (45, 46) and 18 (59, 60) are in LD with r^2^ = 15–67%. For other SNPs within ± 1 Mb flanking region, LD was smaller than 1% (Supplementary Table 2A)*.

*Chr, chromosome; Location, location in base pairs; EA, effect allele; EAF, EA frequency*.

*Column “Trait” shows phenotype with GW significance in the fixed-effect meta-analysis (β_meta_ and p_meta_) and/or Fisher's test (p_Fisher_). ADPN, Adiponectin; AlbU, albumin in urine; AlbS, albumin in serum; CRP, C-reactive protein; HR, heart rate; HDL-C, high-density lipoprotein cholesterol; IL6, interleukin 6; TC, total cholesterol; TG, triglycerides*.

*Column “Ratio”: log (p_meta_)/log(p_Fisher_); I^2^ is the heterogeneity coefficient*.

*^c^Known SNPs were defined as either the index SNP (denoted by letter “S” in column LD) or a SNP with the smallest p-value (p_known_) from GRASP catalog within ± 1 Mb from the index SNP. Numbers in column LD show r^2^ (%) for SNPs in columns “SNP” and “SNP_known_.” Empty cells are for SNPs for which LD could not be evaluated in either our data or in the CEU population from the 1000 Genomes Project*.

*The results for each cohort are provided in Supplementary Table 2B*.

Out-performance of the meta-test by the Fisher test indicates a substantial role of heterogeneity in associations across cohorts (see “Materials and Methods”). This is evidenced by a strong and highly significant inverse correlation of the ratio of log-transformed *p-*values from these two tests, log_10_ (*p*_*meta*_)/log_10_ (*p*_*Fisher*_), with the heterogeneity coefficient I^2^ (Table 2; *r*_*Pearson*_ = −0.874, *p* = 2.0 × 10^−21^). This correlation implies that the larger the *p*_*meta*_ relative to the *p*_*Fisher*_, the more heterogeneous the association signals across cohorts. Forest plots in Supplementary Figure 2 illustrate homogeneous and heterogeneous associations for uncommon SNPs associated with AlbU across cohorts.

The univariate meta-analysis replicated 198 associations for 160 SNPs (68 loci) with *p* < *p*_*GW*_ (Supplementary Table 3, Supplementary Material). For most of these SNPs, *p-*values were smaller for the meta-test than for the Fisher test (*p*_*meta*_ < *p*_*Fisher*_) except for rs1260326, rs13333226, and rs2075650. As for the novel SNPs in Table 2, we observed a strong and highly significant inverse correlation of log_10_ (*p*_*meta*_)/log_10_ (*p*_*Fisher*_) with I^2^ (*r*_*Pearson*_ = −0.603, *p* = 5.5 × 10^−21^). Most of these 198 associations (166 or 83.8%) were observed for lipids.

### Univariate Meta-Analysis and Longitudinal Information

Longitudinal information from repeated measurements benefits univariate analyses for SNPs with relatively homogeneous associations across visits by decreasing standard errors (Supplementary Figure 3). For example, rs17645031 attained *p* = 6.01 × 10^−23^ in our modest sample (Table 2, SNP #4) and *p* = 1.12 × 10^−22^ in a previously described sample of 97,063 individuals (Teslovich et al., 2010) (Supplementary Figure 3C). This improvement substantially increases the efficiency of genetic association studies, which can be quantified by the ratio of the log-transformed *p-*value to the sample size (Kulminski et al., 2016b). For rs17645031, the efficiency can be approximated by the ratio of the sample sizes because the *p-*values in the different studies are virtually the same, yielding a 3.7-fold (= 97,063/26,371) larger efficiency in our study than in the previous study (Teslovich et al., 2010).

### Pleiotropic Meta-Analysis

We used four tests in pathway 1 and three tests in pathway 2 (Figure 1) to examine pleiotropy in six domains: three domains of physiological, blood, and inflammation markers and three domains of all 16 quantitative markers, seven diseases and death, and all 24 phenotypes (Table 1; see “Materials and Methods”). Pleiotropic associations were defined as at least one test showing GW significance.

**Figure 3:**
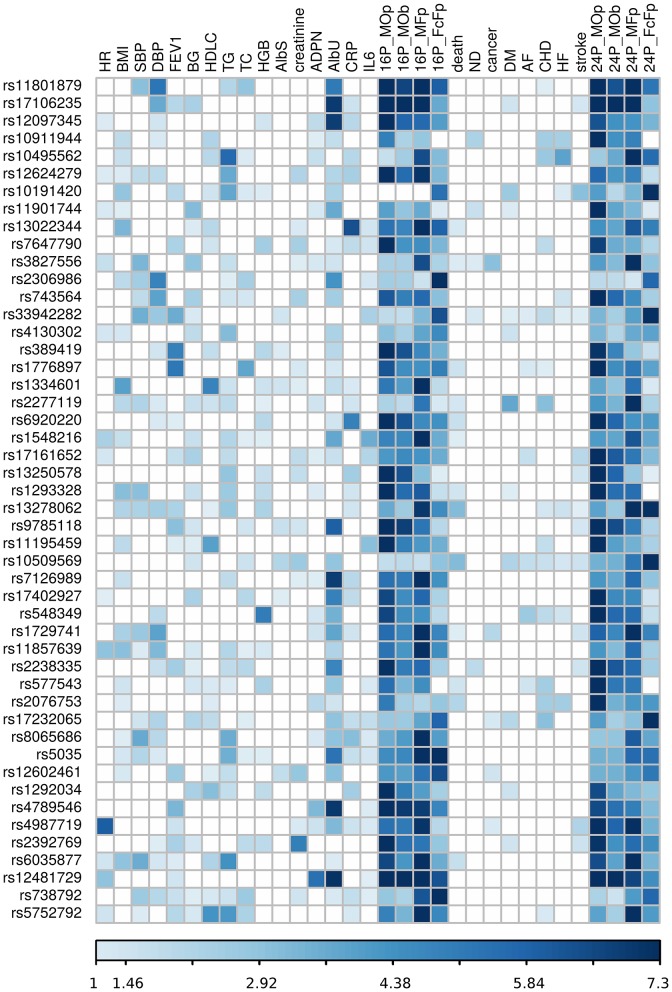
Heat map of phenotype-specific associations for selected pleiotropic SNPs. Data are for SNPs with pleiotropic associations in the domains of 16 quantitative markers (16P) and all 24 phenotypes (24P) from Table 3. Phenotypes are defined in Table 1. FcFp, MFp, MOp, and MOb denote pleiotropic meta-tests (Figure 1). Colors code -log_10_ (*p*-value) trimmed at GW level -log_10_ (5 × 10^−8^) = 7.3 for better resolution.

Our analysis identified 176 novel pleiotropic SNPs (152 loci) with *p*_*pleio*_ < *p*_*GW*_ (Tables 3, 4 and Supplementary Tables 4, 5) by combining associations with multiple phenotypes that individually did not attain GW significance in univariate meta-analysis (*p*_*uni*_ > *p*_*GW*_). Accordingly, this analysis identified SNPs for which associations may be considered “noise” in univariate analyses (Supplementary Figure 4). Of these, 79 (75 loci) and 97 (86 loci) SNPs attained GW significance in pathway 1 (Table 3 and Supplementary Table 4) and pathway 2 (Table 4 and Supplementary Table 5), respectively. Table 5 illustrates these associations for selected SNPs. Most SNPs in each pathway showed associations in the domain of 16 quantitative markers: 29 of 79 SNPs (pathway 1) and 77 of 97 SNPs (pathway 2). The remaining SNPs showed associations in the domains of: (i) 24 phenotypes (19 and 12 in pathways 1 and 2, respectively), (ii) inflammation markers (20 and 4), (iii) blood markers (4 and 4), and (iv) physiological markers (7 in pathway 1 only). No associations with *p* < *p*_*GW*_ were identified in the disease/death domain. The 79 SNPs identified in pathway 1 were associated with up to 11 phenotypes at *p* < 0.05 (Table 3 and Supplementary Table 4), with most associations clustered in the domain of 16 quantitative markers. Figure 3 shows pleiotropic SNPs that attained GW significance in the domains of 16 quantitative markers or 24 phenotypes. Our analysis also identified 11 SNPs in the MHC (Supplementary Table 6).

**Table 3:** Novel pleiotropic SNPs attained genome-wide significance (*p* < 5 × 10^−8^) in pathway 1.

**ID**	**Locus^a^**	**SNP^b^**	**Chr**	**Location, base pairs**	**EA**	**EAF**	**Domain**	**N_**P**_**	**Meta** ***p*****-values**				**Group**	***P*_**GRASP**_**	**N_**G**_**
									**P_**MOp**_**	**P_**MOb**_**	**P_**MFp**_**	**P_**FcFp**_**			
1	NPPB^*^	rs11801879	1	11,868,762	g	0.090	16P	5	6.33E-09	2.04E-07	6.49E-10	1.09E-06	M	5.00E-08	2
2	*FAF1*	rs17106235	1	50,477,698	c	0.098	16P	5	7.42E-09	2.50E-09	3.43E-08	2.13E-04	M	8.70E-03	2
3	GADD45A^*^	rs12097345	1	67,677,857	g	0.063	16P	5	1.23E-08	1.50E-06	2.26E-06	8.30E-05	HP		
4	*LMNA*	rs6686943	1	156,123,200	g	0.024	INF	3	4.95E-07	7.70E-08	1.20E-09	8.47E-10	C		
5	*F5*	rs6025	1	169,549,811	a	0.028	INF	3	2.01E-07	4.74E-08	2.17E-09	4.80E-07	M		
6	*PLA2G4A*	rs10911944	1	186,881,788	g	0.054	24P	6	3.41E-08	3.82E-05	1.21E-05	1.90E-01	HP		
7	*ADAM17/IAH1*	rs10495562	2	9,491,211	a	0.481	24P	7	2.69E-03	1.91E-04	2.35E-09	2.48E-06	D	4.04E-04	2
8	*FOSL2*	rs12624279	2	28,411,923	a	0.406	16P	6	3.59E-08	2.49E-06	8.22E-08	5.33E-04	M	3.41E-06	1
9	*COL5A2*	rs10191420	2	189,159,234	g	0.037	24P	7	8.70E-05	9.02E-03	3.75E-04	4.12E-09	H1a	1.62E-03	3
10	SDPR/TMEFF2^*^	rs11901744	2	191,888,309	g	0.045	24P	3	2.03E-08	1.37E-04	4.41E-04	7.21E-02	HP	2.16E-04	2
11	*TRAK2*	rs13022344	2	201,399,433	g	0.330	16P	4	4.01E-06	1.77E-05	4.69E-08	1.30E-06	D	4.25E-02	1
12	*SSUH2*	rs7647790	3	8,730,403	a	0.022	16P	5	1.83E-08	9.56E-06	8.19E-06	8.15E-04	HP	3.90E-02	1
13	*IRAK2*	rs1681663	3	10,178,045	a	0.233	INF	2	1.94E-07	1.94E-07	1.81E-08	7.68E-08	M		
14	*TFRC*	rs3827556	3	196,069,703	t	0.074	24P	6	8.44E-05	6.83E-04	1.21E-08	1.15E-03	D		
15	*MTTP*	rs2306986	4	99,583,418	g	0.036	16P	6	2.86E-03	3.41E-03	2.58E-02	3.68E-08	H1a	5.84E-06	3
16	*CSF2*	rs743564	5	132,075,186	g	0.393	24P	8	3.64E-08	2.36E-06	2.84E-05	3.71E-03	HP	3.06E-03	2
17	IL13^*^	rs2243297	5	132,663,479	a	0.040	INF	3	3.84E-06	4.74E-07	4.04E-09	2.04E-05	D		
18	ADRB2^*^	rs33942282	5	148,829,255	a	0.028	24P	6	4.85E-04	3.64E-03	1.09E-04	9.05E-09	H1a		
19	*CDKAL1*	rs4130302	6	21,103,928	a	0.022	16P	5	9.27E-04	5.37E-05	2.87E-08	3.12E-04	D	2.60E-02	1
20	UBD^*^	rs389419	6	29,553,668	a	0.088	16P	5	1.82E-09	4.53E-07	1.91E-05	3.43E-04	HP	2.35E-07	5
21	*NRM*	rs1075496	6	30,690,462	a	0.431	PHY	4	9.09E-08	4.88E-09	1.58E-05	2.29E-02	HB		
22	HMGA1^*^	rs1776897	6	34,227,234	c	0.088	24P	4	8.67E-09	3.35E-05	5.56E-06	1.31E-04	HP	7.07E-03	2
23	*MRPS18A*	rs1334601	6	43,685,668	a	0.098	16P	6	6.48E-06	8.65E-05	4.11E-08	1.33E-02	D		
24	*CYP39A1*	rs2277119	6	46,642,168	a	0.233	24P	10	5.57E-05	4.18E-04	7.01E-09	5.29E-03	D	6.68E-04	3
25	TNFAIP3^**^	rs6920220	6	137,685,367	a	0.207	16P	3	3.16E-09	5.38E-07	2.14E-05	3.81E-05	HP	3.80E-03	2
26	*ESR1*	rs2747648	6	152,101,200	g	0.037	INF	3	8.85E-06	1.64E-06	3.49E-08	6.95E-05	D		
27	*IL6*	rs1548216	7	22,730,154	c	0.025	16P	6	1.38E-05	2.46E-05	2.32E-08	2.59E-04	D		
28	IGFBP3^*^	rs1496499	7	45,939,424	c	0.465	PHY	1	1.24E-08	1.18E-07	5.28E-03	2.46E-03	HP	3.06E-02	1
29	*MYH16*	rs17161652	7	99,293,904	g	0.022	24P	9	1.01E-08	8.13E-07	5.72E-06	6.60E-04	HP		
30	*FOXP2*	rs13227011	7	114,614,673	a	0.065	INF	2	5.78E-08	9.39E-08	1.23E-08	3.51E-08	M		
31	*GATA4*	rs13250578	8	11,756,796	a	0.145	16P	4	3.73E-09	4.69E-07	7.99E-04	8.71E-02	HP	1.59E-05	3
32	*FDFT1*	rs1293328	8	11,837,551	a	0.267	16P	6	9.52E-09	2.46E-06	7.97E-07	2.07E-02	HP	4.13E-04	1
33	*TNFRSF10A*	rs13278062	8	23,225,458	c	0.479	24P	10	1.34E-04	2.19E-03	4.55E-10	3.07E-09	C		
34	*IKBKB*	rs9785118	8	42,328,973	g	0.038	16P	6	2.05E-10	1.51E-07	4.78E-06	1.06E-02	HP	3.80E-03	1
35	*PCSK5*	rs7022503	9	76,127,438	g	0.491	BLD	4	1.17E-03	1.19E-04	3.73E-08	2.99E-04	D	2.75E-04	2
36	*IKBKAP*	rs4978374	9	108,884,703	a	0.239	PHY	3	1.38E-01	1.02E-01	2.51E-01	3.95E-08	H1a	2.53E-07	2
37	GATA3^**^	rs10508372	10	8,930,055	a	0.075	BLD	5	6.37E-05	3.75E-06	1.09E-09	3.80E-08	C	2.18E-05	3
38	*CXCL12*	rs266092	10	44,370,827	a	0.047	INF	3	1.07E-06	3.23E-07	1.03E-08	1.40E-06	D		
39	*NRG3*	rs11195459	10	82,660,517	g	0.054	16P	3	1.48E-09	9.70E-06	5.99E-05	2.87E-04	HP	1.47E-02	1
40	*LIPA*	rs10509569	10	89,221,893	a	0.064	24P	11	3.29E-04	3.21E-04	6.44E-06	4.36E-08	H1a	1.83E-03	2
41	*PKD2L1*	rs735877	10	100,344,764	a	0.383	PHY	3	1.35E-05	2.05E-05	4.16E-08	1.04E-04	D	7.08E-04	3
42	*CYP2E1*	rs7081484	10	133,538,986	a	0.025	INF	3	1.84E-07	4.28E-08	2.00E-09	2.58E-08	M		
43	LRRC4C^**^	rs7126989	11	42,152,027	a	0.027	16P	4	3.73E-06	7.74E-06	3.29E-08	3.77E-05	D		
44	*RAD9A*	rs2255990	11	67,397,024	a	0.034	INF	2	4.55E-07	1.23E-07	5.48E-09	9.45E-08	M		
45	*CPT1A*	rs11228373	11	68,829,752	g	0.128	BLD	3	1.50E-09	2.61E-05	5.47E-05	1.12E-02	HP	5.05E-03	2
46	*SOX5*	rs17402927	12	24,020,644	g	0.027	24P	4	1.01E-08	2.24E-05	2.30E-05	3.63E-02	HP		
47	*HDAC7*	rs17122311	12	47,795,115	a	0.027	INF	2	5.30E-08	5.04E-08	8.54E-08	8.60E-07	M		
48	*APAF1*	rs4319556	12	98,682,808	a	0.026	INF	3	9.52E-08	1.54E-08	2.41E-10	1.28E-07	M		
49	HCAR1/DENR^*^	rs548349	12	122,740,362	a	0.065	24P	7	2.36E-09	2.24E-06	2.51E-06	2.01E-02	HP		
50	*BRCA2*	rs11571590	13	32,320,347	a	0.029	INF	3	1.50E-06	4.89E-07	5.39E-08	8.84E-09	H1a		
51	*FOXO1*	rs9603776	13	40,649,749	a	0.022	INF	2	6.61E-05	1.66E-04	3.15E-04	3.12E-08	H1a		
52	*ABCC4*	rs1729741	13	95,131,902	g	0.116	16P	6	2.72E-06	1.85E-05	1.97E-08	1.74E-05	D	2.60E-02	1
53	*IRS2*	rs1044364	13	109,755,299	g	0.049	PHY	3	4.85E-05	1.07E-04	1.87E-08	2.05E-05	D	1.82E-02	1
54	*TEP1*	rs10083536	14	20,408,030	a	0.030	INF	2	2.89E-07	7.37E-08	4.66E-09	2.05E-10	C		
55	MDGA2^**^	rs17117423	14	46,539,309	g	0.039	INF	3	5.84E-07	3.00E-07	6.78E-09	3.22E-07	D		
56	*FBN1*	rs1848050	15	48,569,846	a	0.102	INF	3	2.80E-05	4.06E-06	4.59E-08	1.24E-05	D		
57	*CYP19A1*	rs28757184	15	51,222,375	a	0.034	INF	2	4.05E-08	3.09E-07	4.42E-07	2.65E-06	HP		
58	*HCN4*	rs11857639	15	73,345,431	a	0.092	16P	5	4.20E-06	5.52E-05	4.58E-08	3.17E-05	D	6.39E-03	1
59	HCN4^*^	rs7172808	15	73,371,291	g	0.161	PHY	2	1.68E-07	4.24E-08	2.40E-05	1.41E-04	HB	3.16E-02	1
60	*BLM*	rs2238335	15	90,747,136	c	0.024	24P	7	8.67E-09	7.30E-07	2.76E-06	2.98E-03	HP	4.40E-03	2
61	*LMF1*	rs577543	16	888,967	a	0.073	24P	7	3.03E-08	5.64E-06	5.95E-06	2.17E-01	HP	1.25E-04	3
62	*NOD2*	rs2076753	16	50,699,463	a	0.290	24P	5	3.99E-08	3.31E-04	6.42E-05	6.26E-05	HP	4.40E-02	1
63	MMP2/IRX6^*^	rs17232065	16	55,391,471	a	0.067	24P	9	9.60E-05	3.47E-03	9.92E-04	6.74E-09	H1a	2.14E-02	1
64	*PYY*	rs9895585	17	43,987,619	c	0.049	INF	3	2.28E-07	4.84E-08	1.62E-09	1.38E-07	M		
65	*HDAC5*	rs228757	17	44,087,517	c	0.266	PHY	3	1.11E-05	1.59E-05	4.54E-08	2.27E-04	D	6.41E-04	2
66	*HDAC5*	rs8065686	17	44,116,529	a	0.093	16P	5	2.45E-04	7.70E-05	4.47E-09	8.62E-05	D	1.35E-03	1
67	*SLC4A1*	rs5035	17	44,261,630	c	0.040	16P	6	2.53E-05	2.02E-05	1.06E-08	3.03E-08	C	8.72E-03	2
68	*OSBPL7*	rs12602461	17	47,811,983	a	0.052	16P	4	6.57E-05	1.64E-06	3.63E-08	5.62E-08	C	9.14E-04	2
69	*RPS6KB1*	rs1292034	17	59,912,499	a	0.445	16P	5	1.16E-08	1.07E-05	9.55E-05	3.81E-03	HP		
70	*APOH*	rs1801689	17	66,214,462	c	0.034	BLD	3	1.19E-09	3.26E-09	2.93E-04	3.87E-02	HP	4.92E-06	1
71	*SYNGR2*	rs4789546	17	78,171,312	a	0.045	16P	3	2.81E-08	1.02E-07	1.30E-07	1.78E-05	M		
72	*BCL2*	rs10164240	18	63,277,305	g	0.022	INF	3	9.57E-07	3.38E-07	2.10E-08	2.51E-06	D		
73	*BCL2*	rs4987719	18	63,293,077	a	0.032	24P	6	1.97E-08	1.51E-06	2.59E-08	3.48E-04	M	3.70E-03	1
74	LMNB2^*^	rs2392769	19	2,461,807	g	0.237	16P	5	3.56E-09	4.68E-06	5.15E-06	1.57E-03	HP		
75	NKX2-2^*^	rs6035877	20	21,531,894	c	0.474	16P	6	2.28E-07	5.30E-05	1.66E-08	2.25E-04	D	1.72E-05	4
76	*APP*	rs17001660	21	26,051,722	g	0.051	INF	2	4.24E-04	2.96E-04	8.05E-05	2.22E-08	H1a		
77	*APP*	rs12481729	21	26,148,455	c	0.025	16P	4	1.47E-11	4.61E-10	4.09E-09	4.79E-07	M		
78	*MMP11*	rs738792	22	23,779,191	g	0.085	16P	7	1.51E-03	5.00E-03	6.14E-07	6.40E-09	H1a		
79	*HSCB*	rs5752792	22	28,757,771	g	0.190	16P	6	1.23E-05	4.09E-04	3.73E-09	2.03E-05	D	4.80E-05	3

a *If an index SNP was not within protein coding gene, the closest gene was assigned. Multiple genes were assigned if they were at about the same distance up- and downstream from the index SNP or if the index SNP was within the region of overlapping genes. Loci were naturally associated with genes*.

* *Protein coding genes within ±100 Kb flanking region for the index SNP*.

** *Protein coding genes for four SNPs (#25, 37, 43, and 55) are within ± 1 Mb flanking region for the index SNP*.

b *Proxy SNPs with linkage disequilibrium (LD) r^2^ > 70% were excluded. LD for SNPs within ± 1 Mb flanking region retained in the table is given in Supplementary Table 4A*.

*Chr, chromosome; EA, effect allele; EAF, EA frequency*.

*Domain indicates the pleiotropic domain in which minimal GW significant p-values were attained in our pleiotropic meta-analysis examining: physiological (PHY), blood (BLD), inflammation (INF), 16 quantitative markers (16P), 7 diseases and death (8DD), and all 24 phenotypes (24P) domains. P-values are given for all four pleiotropic meta-tests (FcFp, MFp, MOp, and MOb) for a given domain (Figure 1E)*.

*Group indicates the type of association defined in the “Genetic heterogeneity and correlation in pleiotropic meta-analysis” section*.

*P_GRASP_ shows p-values from the pleiotropic meta-analysis of results for the index SNPs reported in GRASP*.

*N_P_ and N_G_ show the number of phenotypes that attained a minimal p-value (p < 0.05) in the meta- or Fisher test in a given phenotypic domain in pathway 1 and Fisher test of GRASP results, respectively*.

*More details on the associations for these pleiotropic SNPs are given in Supplementary Tables 4B–D*.

**Table 4:** Novel pleiotropic SNPs attained genome-wide significance (*p* < 5 × 10^−8^) in pathway 2.

**ID**	**Locus^a^**	**SNP^b^**	**Chr**	**Location, base pairs**	**EA**	**EAF**	**Domain**	**Meta** ***p*****-values**			**Group**	***P*_**GRASP**_**	**N_**G**_**
								**P_**OpFc**_c**	**P_**ObFc**_c**	**P_**FpFc**_c**			
1	*HTR6*	rs3790756	1	19,669,739	a	0.136	16P	6.28E-09	6.30E-04	2.19E-04	HP	1.24E-03	2
2	*PODN*	rs17107806	1	53,066,863	c	0.023	16P	1.49E-08	6.51E-04	2.00E-03	HP		
3	*PDE4B*	rs6667596	1	66,320,317	g	0.065	16P	8.94E-12	2.02E-06	8.76E-06	HP	4.92E-02	1
4	*PDE4B*	rs6696880	1	66,335,812	g	0.062	16P	4.46E-09	1.95E-05	7.82E-05	HP	1.19E-04	4
5	*ABCA4*	rs10874828	1	94,004,017	a	0.095	16P	3.20E-08	2.02E-04	4.89E-04	HP		
6	*VCAM1*	rs3917011	1	100,726,586	g	0.027	16P	3.36E-08	2.80E-05	9.17E-07	M		
7	*ARNT*	rs10305670	1	150,858,612	c	0.021	16P	1.63E-11	2.23E-05	1.27E-05	HP		
8	*SHC1*	rs12076073	1	154,971,680	g	0.038	24P	1.24E-04	1.07E-04	3.30E-08	C		
9	*NOS1AP*	rs2661808	1	162,302,581	c	0.022	16P	8.79E-09	1.75E-04	3.06E-03	HP	1.08E-02	2
10	*PARP1*	rs3219123	1	226,367,647	a	0.062	24P	3.24E-08	4.56E-04	6.75E-04	HP		
11	*NLRP3*	rs4925659	1	247,440,161	a	0.384	24P	2.32E-08	2.03E-04	1.48E-05	HP	5.99E-03	2
12	CXCR4^*^	rs9973445	2	136,121,046	c	0.103	16P	3.36E-08	7.61E-05	3.05E-04	HP	1.22E-02	1
13	*NEB/RIF1*	rs1061305	2	151,490,465	g	0.410	16P	1.03E-08	5.40E-04	7.50E-03	HP	4.18E-04	4
14	*TFPI*	rs8176595	2	187,504,645	a	0.030	16P	2.54E-10	8.68E-06	3.06E-05	HP	3.14E-02	1
15	SLC39A10^*^	rs6748661	2	194,818,116	a	0.142	16P	7.91E-10	7.02E-06	1.56E-06	HP		
16	*INPP5D*	rs6715810	2	233,164,597	a	0.172	16P	1.34E-09	5.68E-05	1.86E-03	HP	7.09E-05	2
17	*INPP5D*	rs6725722	2	233,164,999	a	0.038	16P	3.84E-08	1.06E-04	1.19E-05	HP	1.27E-02	1
18	*CAV3/SSUH2*	rs6764715	3	8,744,886	a	0.026	INF	3.05E-07	1.53E-07	1.31E-08	C		
19	*IRAK2*	rs713016	3	10,237,012	a	0.126	16P	3.86E-08	5.72E-04	1.86E-04	HP		
20	*SCN5A*	rs7624535	3	38,623,711	c	0.215	16P	1.92E-08	3.87E-03	1.20E-01	HP		
21	*GNAI2*	rs762707	3	50,252,421	a	0.023	INF	2.44E-08	3.12E-08	5.11E-08	M		
22	*EVC*	rs2291157	4	5,719,294	c	0.086	16P	4.31E-09	1.68E-05	1.52E-06	HP		
23	*EVC*	rs7674034	4	5,741,326	g	0.338	16P	5.47E-08	9.88E-05	3.94E-08	CB	8.78E-04	2
24	IL21^*^	rs17005953	4	122,635,086	a	0.054	24P	3.34E-08	4.48E-05	1.76E-05	HP	2.72E-04	2
25	*IL15*	rs7698675	4	141,673,566	t	0.264	16P	6.77E-10	5.15E-06	1.05E-05	HP	3.62E-02	1
26	*SLC9A3*	rs6864158	5	505,936	g	0.423	16P	4.84E-08	2.54E-04	1.53E-03	HP	3.32E-03	2
27	*SSBP2*	rs6452419	5	81,575,583	a	0.323	16P	3.76E-08	1.01E-03	8.16E-04	HP	7.00E-03	1
28	*ADGRV1*	rs2438353	5	90,770,429	a	0.482	16P	5.58E-09	6.53E-04	7.42E-03	HP	1.29E-03	2
29	*ADGRV1*	rs7712313	5	90,932,581	g	0.309	24P	7.50E-08	2.40E-06	4.44E-08	CB	3.53E-05	4
30	*ADGRV1*	rs16869425	5	91,089,281	c	0.104	16P	2.29E-08	3.06E-04	6.93E-04	HP	2.69E-04	4
31	*SLCO4C1*	rs10066650	5	102,251,670	c	0.105	16P	4.12E-08	4.44E-05	3.12E-05	HP		
32	*FBN2*	rs3805652	5	128,425,725	g	0.135	16P	8.16E-09	3.06E-04	7.68E-03	HP	5.03E-03	2
33	GMNN^*^	rs6904263	6	24,771,241	a	0.181	16P	4.40E-04	1.17E-02	2.45E-08	C	2.63E-03	2
34	TREM2^*^	rs7759295	6	41,168,112	a	0.124	16P	2.39E-08	1.60E-04	1.17E-03	HP	2.11E-03	2
35	PKHD1^*^	rs10948623	6	51,553,432	a	0.219	16P	2.55E-08	3.28E-03	1.24E-03	HP	5.28E-04	2
36	*PDSS2*	rs12199523	6	107,187,066	g	0.199	16P	2.46E-09	4.07E-05	1.18E-06	HP		
37	*ARG1/MED23*	rs17788484	6	131,573,218	a	0.023	16P	7.35E-11	2.44E-06	9.60E-05	HP		
38	*MTHFD1L*	rs2073188	6	150,937,175	a	0.063	16P	7.36E-09	1.01E-04	9.20E-05	HP		
39	*MTHFD1L*	rs9478918	6	151,037,163	a	0.139	16P	2.36E-08	5.56E-05	3.73E-05	HP	2.47E-03	3
40	*PLG*	rs9456577	6	160,724,214	c	0.028	16P	2.68E-08	1.00E-04	3.11E-05	HP	2.06E-03	2
41	*IGFBP3*	rs3793345	7	45,918,079	g	0.204	16P	3.35E-08	1.15E-04	1.07E-02	HP		
42	*HGF*	rs5745692	7	81,728,950	g	0.033	BLD	3.26E-08	4.30E-05	4.12E-04	HP		
43	*CDK6*	rs17690388	7	92,716,043	a	0.052	16P	7.42E-10	1.44E-05	2.36E-05	HP	8.11E-03	2
44	*MSRA*	rs7840347	8	10,214,840	g	0.439	16P	1.15E-08	4.34E-05	2.03E-05	HP	3.24E-06	5
45	*DLC1*	rs1442534	8	13,117,098	a	0.168	16P	3.26E-08	3.39E-03	7.66E-04	HP		
46	STMN4 112kb^**^	rs11135979	8	27,123,055	a	0.040	16P	6.30E-09	1.26E-05	1.35E-07	M	9.90E-03	1
47	PLAT^*^	rs2020918	8	42,214,920	a	0.341	24P	2.30E-08	4.15E-05	4.88E-09	CB	9.90E-03	1
48	*TERF1*	rs10104094	8	73,016,217	a	0.455	16P	1.14E-12	2.27E-06	7.81E-07	HP		
49	*MMP16*	rs10103111	8	88,062,998	a	0.223	16P	3.99E-08	1.48E-04	1.74E-04	HP	9.22E-03	2
50	*SDC2*	rs2464474	8	96,585,444	g	0.110	16P	1.97E-11	1.83E-03	9.71E-03	HP	4.68E-02	1
51	SCRT1/TMEM249^*^	rs4333645	8	144,345,873	a	0.433	16P	2.31E-08	1.56E-04	1.19E-04	HP	4.96E-05	1
52	*CA9*	rs7858447	9	35,677,248	g	0.192	16P	1.62E-09	1.64E-05	1.23E-07	HP		
53	*C9orf114/ENDOG*	rs2280845	9	128,820,891	a	0.249	24P	2.51E-09	1.83E-05	5.32E-04	HP	5.77E-07	2
54	*PTGES*	rs7872802	9	129,753,451	g	0.136	16P	3.34E-06	3.11E-05	4.37E-08	C	2.58E-02	1
55	RXRA^*^	rs36123461	9	134,441,548	g	0.020	16P	3.64E-09	8.30E-05	2.05E-04	HP		
56	*OIT3*	rs2394931	10	72,906,307	a	0.055	16P	9.90E-09	2.03E-04	1.30E-04	HP	2.92E-02	1
57	*VCL*	rs2395075	10	74,008,422	g	0.251	16P	2.95E-08	1.20E-03	5.92E-04	HP	5.32E-03	1
58	*NRG3*	rs1649960	10	82,273,509	g	0.065	16P	3.15E-08	4.85E-05	6.36E-05	HP	2.57E-02	1
59	*NRG3*	rs10509455	10	82,507,966	c	0.101	24P	1.30E-08	4.67E-04	9.69E-04	HP		
60	*GRK5*	rs2230349	10	119,436,823	a	0.100	16P	3.19E-09	3.58E-05	6.89E-05	HP		
61	*LSP1*	rs2089910	11	1,853,174	a	0.235	INF	2.42E-08	1.72E-07	5.59E-07	M		
62	LRRC4C 110 kb^**^	rs1457326	11	41,573,519	a	0.242	16P	1.40E-08	1.40E-03	5.79E-04	HP	1.68E-02	1
63	*NOX4*	rs553635	11	89,496,263	a	0.074	16P	1.90E-08	9.94E-06	2.00E-05	HP	6.43E-04	3
64	*MMP10*	rs17359286	11	102,772,987	a	0.037	24P	2.16E-08	4.35E-05	1.55E-06	HP	1.52E-02	2
65	*BCAT1*	rs7964239	12	24,920,751	g	0.082	16P	3.07E-08	5.54E-04	1.39E-03	HP	6.43E-04	3
66	*HDAC7*	rs2301783	12	47,788,458	c	0.123	16P	4.54E-09	4.64E-05	3.48E-06	HP	1.75E-02	1
67	*ATF1*	rs3742065	12	50,795,836	g	0.051	16P	4.39E-08	2.32E-05	1.76E-05	HP	1.90E-03	2
68	*RARG*	rs10082919	12	53,225,680	a	0.045	16P	1.25E-08	9.51E-05	2.81E-04	HP		
69	*IRS2*	rs7999797	13	109,773,653	g	0.449	24P	4.83E-08	1.59E-05	9.82E-05	HP	7.64E-04	3
70	*MTHFD1*	rs17824591	14	64,420,993	a	0.213	16P	2.51E-08	5.13E-05	3.02E-07	M		
71	*SERPINA1*	rs1980618	14	94,386,086	t	0.355	16P	3.37E-09	9.48E-04	9.92E-03	HP	3.72E-02	1
72	EXOC3L4/TNFAIP2^*^	rs7155575	14	103,116,465	a	0.1524	16P	3.02E-08	5.43E-04	5.25E-04	HP	4.13E-02	1
73	BAG5^*^	rs10129426	14	103,552,118	g	0.462	16P	8.47E-10	9.88E-04	2.40E-04	HP	3.88E-04	1
74	*C15orf41*	rs8039329	15	36,658,364	a	0.129	16P	3.14E-08	2.85E-04	2.37E-03	HP	9.69E-03	2
75	*PCSK6*	rs8042699	15	101,386,868	a	0.331	16P	1.11E-08	5.95E-05	2.32E-06	HP	3.21E-03	2
76	HBA2^*^	rs2974771	16	171,058	a	0.471	16P	1.89E-08	1.29E-03	3.53E-04	HP	3.90E-02	1
77	*ACSM3/EXOD1*	rs5716	16	20,785,065	c	0.084	16P	2.72E-08	1.79E-05	5.61E-05	HP		
78	*GAS7*	rs9545	17	9,910,885	g	0.058	16P	1.14E-09	1.26E-04	5.90E-04	HP	2.54E-02	1
79	SEPT9 176kb^**^	rs7218173	17	77,676,394	c	0.075	16P	1.31E-08	6.95E-06	4.35E-07	HP	3.72E-02	1
80	*SOCS3*	rs4969168	17	78,357,712	a	0.140	16P	1.76E-09	3.29E-05	2.24E-03	HP	1.81E-05	4
81	*BCL2*	rs2046135	18	63,147,019	a	0.033	BLD	1.62E-08	7.42E-07	4.19E-06	HP		
82	*BCL2*	rs17070739	18	63,152,150	c	0.038	16P	1.20E-09	2.86E-06	3.00E-05	HP	3.90E-02	1
83	*BCL2*	rs8097624	18	63,153,066	a	0.022	BLD	5.88E-09	2.87E-07	3.95E-05	HP		
84	*C3*	rs11666133	19	6,686,410	a	0.043	16P	9.13E-06	8.29E-04	2.18E-08	C	4.31E-02	1
85	LRRC25^*^	rs8101249	19	18,391,020	c	0.176	16P	2.32E-09	1.98E-03	2.92E-04	HP	3.81E-03	2
86	*DMPK*	rs16939	19	45,772,798	a	0.459	16P	4.25E-08	4.29E-05	3.88E-05	HP	1.12E-02	1
87	IGFL4^*^	rs10500295	19	46,072,145	a	0.028	24P	1.61E-08	3.40E-04	2.46E-03	HP	4.44E-02	1
88	*SLC8A2*	rs11083878	19	47,436,586	g	0.083	16P	3.29E-09	2.17E-04	3.69E-03	HP		
89	*BPI*	rs5741804	20	38,323,940	a	0.047	16P	1.81E-10	5.39E-05	5.77E-06	HP	4.20E-02	1
90	*MMP9*	rs9509	20	46,016,514	g	0.024	16P	1.37E-08	4.39E-05	1.67E-06	HP		
91	*NFATC2*	rs6067766	20	51,423,549	a	0.115	16P	1.02E-08	2.82E-04	5.63E-04	HP		
92	*APP*	rs743532	21	26,066,185	g	0.059	INF	1.33E-08	7.68E-08	8.31E-08	M		
93	ABCG1^*^	rs4148082	21	42,199,305	a	0.090	24P	3.43E-05	5.83E-03	1.86E-09	C	1.27E-02	2
94	*ABCG1*	rs3788010	21	42,295,912	g	0.411	BLD	3.48E-08	8.45E-06	4.15E-07	M		
95	VPREB3/C22orf15^*^	rs6003875	22	23,759,673	g	0.237	16P	1.92E-08	2.04E-05	1.44E-06	HP		
96	*PPARA*	rs4253721	22	46,212,338	g	0.045	16P	1.25E-08	2.35E-04	1.43E-04	HP		
97	*PPARA*	rs4253778	22	46,234,737	c	0.184	16P	7.10E-09	4.53E-04	3.71E-03	HP	1.65E-05	2

a *If an index SNP was not within protein coding gene, the closest gene were assigned. Multiple genes were assigned if they were at about the same distance up- and downstream from the index SNP or if the index SNP was within the region of overlapping genes. Loci were naturally associated with genes*.

* *Protein coding genes within ± 100 Kb flanking region for the index SNP*.

** *Protein coding genes for three SNPs (#46, 62, and 79) are within ±200 Kb flanking region for the index SNP*.

b *Proxy SNPs with linkage disequilibrium (LD) r^2^>70% were excluded. LD for SNPs within ±1 Mb flanking region retained in the table is given in Supplementary Table 5A*.

*Chr, chromosome; EA, effect allele; EAF, EA frequency*.

*Domain indicates the pleiotropic domain in which minimal GW significant p-values were attained in our pleiotropic meta-analysis examining: physiological (PHY), blood (BLD), inflammation (INF), 16 quantitative markers (16P), 7 diseases and death (8DD), and all 24 phenotypes (24P) domains. P-values are given for all three pleiotropic meta-tests (FpFc, OpFc, and ObFc) for a given domain (Figure 1D)*.

*Group indicates the type of association defined in the “Genetic heterogeneity and correlation in pleiotropic meta-analysis” section*.

*P_GRASP_ shows p-values from the pleiotropic meta-analysis of results for the index SNPs reported in GRASP*.

*N_G_ show the number of phenotypes that attained a minimal p-value (p < 0.05) in the Fisher test of GRASP results*.

*More details on the associations for these pleiotropic SNPs are given in Supplementary Table 5B*.

**Table 5:** Selected novel pleiotropic genome-wide significant SNPs.

**ID**	**Gene**	**SNP**	**Chr**	**Location base pairs**	**EA**	**EAF**	**Domain**	**N_**P**_**	**Meta** ***p*****-values**				**Group**	***P*_**GRASP**_**	**N_**G**_**
									**MOp/OpFc**	**MOb/ObFc**	**MFp**	**FcFp/FpFc**			
**PATHWAY 1**															
9	*COL5A2*	rs10191420	2	189,159,234	g	0.037	24P	7	8.70E-05	9.02E-03	3.75E-04	4.12E-09	H1a	1.62E-03	3
21	*NRM*	rs1075496	6	30,690,462	a	0.431	PHY	4	9.09E-08	4.88E-09	1.58E-05	2.29E-02	HB		
24	*CYP39A1*	rs2277119	6	46,642,168	a	0.233	24P	10	5.57E-05	4.18E-04	7.01E-09	5.29E-03	D	6.68E-04	3
33	*TNFRSF10A*	rs13278062	8	23,225,458	c	0.479	24P	10	1.34E-04	2.19E-03	4.55E-10	3.07E-09	C		
70	*APOH*	rs1801689	17	66,214,462	c	0.034	BIO	3	1.19E-09	3.26E-09	2.93E-04	3.87E-02	HP	4.92E-06	1
73	*BCL2*	rs4987719	18	63,293,077	a	0.032	24P	6	1.97E-08	1.51E-06	2.59E-08	3.48E-04	M	3.70E-03	1
**PATHWAY 2**															
29	*ADGRV1*	rs7712313	5	90,932,581	g	0.309	24P		7.50E-08	2.40E-06		4.44E-08	CB	3.53E-05	4
54	*PTGES*	rs7872802	9	129,753,451	g	0.136	16P		3.34E-06	3.11E-05		4.37E-08	C	2.58E-02	1
59	*NRG3*	rs10509455	10	82,507,966	c	0.101	24P		1.30E-08	4.67E-04		9.69E-04	HP		
92	*APP*	rs743532	21	26,066,185	g	0.059	INF		1.33E-08	7.68E-08		8.31E-08	M		

*Chr, chromosome; EA, effect allele; EAF, EA frequency*.

*Domain indicates the pleiotropic domain in which minimal GW significant p-values were attained examining: physiological (PHY), blood (BLD), inflammation (INF), 16 quantitative markers (16P), 7 diseases and death (8DD), and all 24 phenotypes (24P) domains. P-values are given for all pleiotropic meta-tests for a given domain (Figures 1D,E)*.

*Group indicates the type of association defined in the “Genetic heterogeneity and correlation in pleiotropic meta-analysis” section*.

*P_GRASP_ shows p-values from the pleiotropic meta-analysis of results for the index SNPs reported in GRASP*.

*N_P_ and N_G_ show the number of phenotypes that attained a minimal p-value (p < 0.05) in the meta- or Fisher test in a given phenotypic domain in pathway 1 and Fisher test of GRASP results, respectively*.

*ID numbers correspond to those in Tables 3, 4 and Supplementary Tables 4, 5 (Supplementary Material) with details on the associations for all novel pleiotropic SNPs*.

Because *p*_*pleio*_ < *p*_*GW*_ < *p*_*uni*_ for pleiotropic SNPs, this inequality automatically validates pleiotropy for 79 SNPs in pathway 1, as it implies that pleiotropic statistics improved to attain *p*_*pleio*_ < *p*_*GW*_ by pooling contributions from multiple phenotypes with *p*_*uni*_ > *p*_*GW*_ (Supplementary Tables 4B–D). Because combining statistics in pathway 2 may yield *p*_*pleio*_ < *p*_*GW*_ driven by strong pleiotropy in one cohort only, the set of 97 SNPs did not include SNPs with *p*_*pleio*_ < *p*_*GW*_ in one cohort that did not attain nominal significance (adjusted for six tests, *p* > 0.05/6 = 8.3 × 10^−3^) in at least one additional cohort (Supplementary Table 7).

### Genetic Heterogeneity and Correlation in Pleiotropic Meta-Analysis

The Fisher test in pathways 1b and 2a (Figure 1) combined *p-*values across phenotypes assuming that they were from independent associations. Given the evolutionary uncertainty in establishing molecular mechanisms of age-related phenotypes, these associations may or may not be independent, even for correlated phenotypes, because the mechanisms driving genetic associations with these phenotypes and the correlations among them may have different origins (see “Materials and Methods”). Then, differences in *p-*values from the Fisher and omnibus tests (Figures 1D,E) reflect the impacts of heterogeneity and/or correlation in genetic associations. Below, we characterize these impacts. We used an *ad-hoc* cut-off for the difference in *p-*values between these tests of ≥1.5 orders of magnitude to characterize a strong impact.

Our analysis identified conventional and unconventional sets of associations in each pathway. The conventional set, represented by three groups of SNPs in pathways 1 (D, C, and M) and 2 (C, CB, and M) (Table 5), was characterized by associations commonly expected in GWAS. It included 43 SNPs from pathway 1 (Table 3) and 16 SNPs from pathway 2 (Table 4), covering 33.5% (59 of 176) of SNPs. The C (6 SNPs in each pathway) and CB (3 SNPs in pathway 2 only) groups included 15 SNPs with *p*<*p*_*GW*_ in the Fisher-based tests and substantially larger (≥1.5 orders of magnitude) *p*-values in the omnibus tests (e.g., *p*_*FcFp*_, *p*_*MFp*_ < *p*_*GW*_ < *p*_*MOp*_, *p*_*MOb*_ for pathway 1). Although this is a commonly expected result for correlated phenotypes, indicating a strong impact of correlation between phenotypes and/or association signals, it was relevant to only 8.5% of SNPs. The difference between the C and CB groups is that for the latter group there was a substantial difference between *p*_*FpFc*_ and *p*_*ObFc*_ but not between *p*_*FpFc*_ and *p*_*OpFc*_. Group D included 23 SNPs (13.1%) with GW significance attained only in the MFp test (i.e., *p*_*MFp*_ < *p*_*GW*_ < *p*_*FcFp*_, *p*_*MOp*_, *p*_*MOb*_). This result indicates SNPs with more homogeneous associations in pathway 1a when *p*_*meta*_ is less than or approximately the same as *p*_*Fisher*_ (Supplementary Tables 4C,D). Group M (14 and 7 SNPs in pathways 1 and 2, respectively) was characterized by attaining GW significance in several tests and/or minor (<1.5 orders of magnitude) differences between *p*-values in these tests.

**Figure 4:**
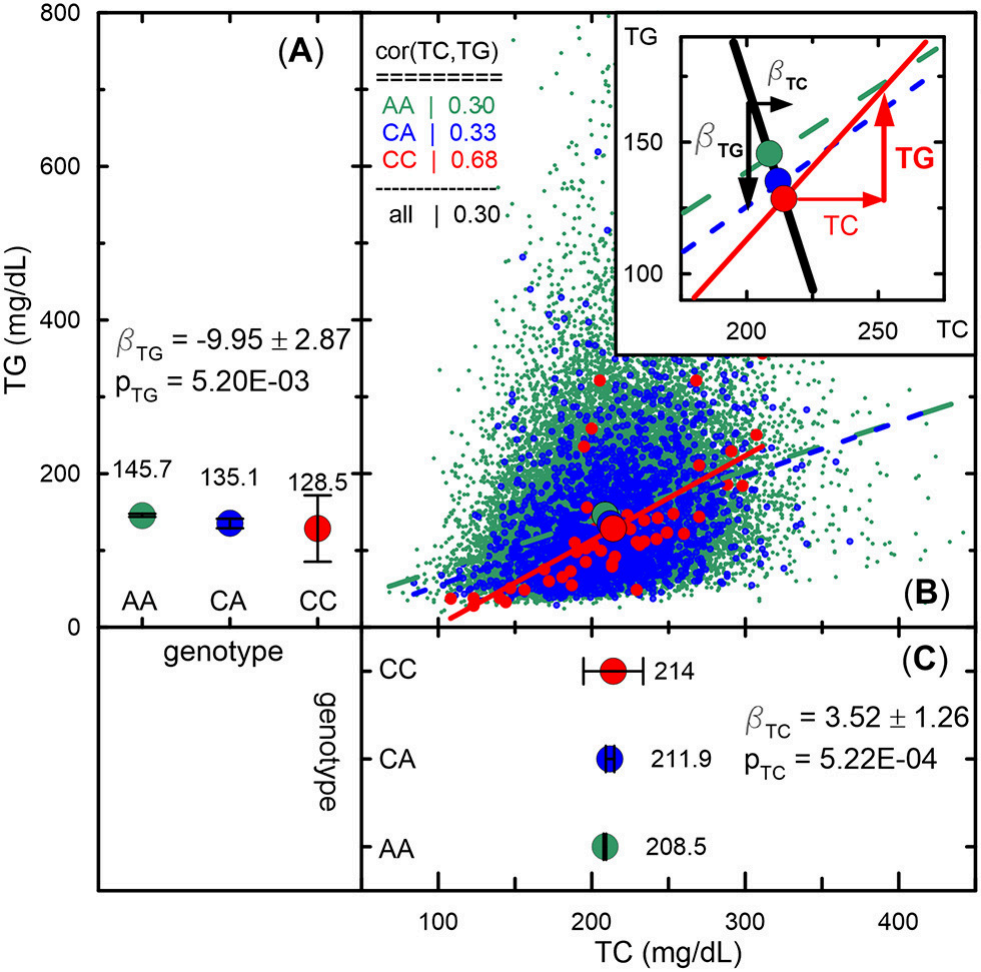
Empirical illustration of antagonistic genetic heterogeneity. **(A,C)** Mean values and 95% confidence intervals of TG and TC, respectively, for rs1801689 genotypes (green for AA, blue for CA, and red for CC) in the Atherosclerosis Risk in Communities Study measured at baseline. Beta and *p* denote effect sizes and *p*-values, respectively. Antagonistic genetic heterogeneity is evidenced by opposite directions of changes in mean values across genotypes for directly correlated phenotypes (i.e., a minor allele associated with a larger concentration of TC is associated with a smaller concentration of TG, whereas a larger concentration of TC correlates [*r* = 0.68 for CC genotype] with a larger concentration of TG for this allele). **(B)** Scatter plot of TG vs. TC. The slopes of lines represent correlation coefficients given in the upper left corner inset. The upper right corner inset illustrates antagonistic genetic heterogeneity by showing opposite directions of genetic effect for TG (black line) and projection of the vector of the correlation on TG (red line). See other details in see “Materials and Methods” section and Figure 2.

The unconventional set, represented by three groups of pleiotropic SNPs in pathway 1 (H1a, HP, and HB) and one group (HP) in pathway 2 (Table 5), was characterized by associations with a strong impact of the evolutionary uncertainty in establishing molecular mechanisms of age-related phenotypes. It included 36 SNPs from pathway 1 (Table 3) and 81 SNPs from pathway 2 (Table 4), covering 66.5% (117 of 176) of SNPs, which defined heterogeneous pleiotropy. The HP group, the most common in each pathway (24 and 81 SNPs in pathways 1 and 2, respectively, covering 59.7% of SNPs), was characterized by attaining GW significance in the **Σ**^***P***^**-**based omnibus tests (Figure 1) and substantially larger (≥1.5 orders of magnitude) *p*-values in the Fisher-based tests (e.g., *p*_*MOp*_ < *p*_*GW*_ < *p*_*MFp*_ for pathway 1). The HB group (2 SNPs) resembled the HP group except for having *p*_*MOb*_ < *p*_*GW*_ instead of *p*_*MOp*_ < *p*_*GW*_. These results indicate a strong impact of antagonistic genetic heterogeneity (Figures 2D, 4) characteristic for 107 SNPs (60.8%). Lastly, the H1a group (10 SNPs) was characterized by attaining GW significance in the FcFp test and substantially larger (≥1.5 orders of magnitude) *p*-values in the other three tests in pathway 1 (i.e., *p*_*FcFp*_ < *p*_*GW*_ < *p*_*MFp*_, *p*_*MOp*_, *p*_*MOb*_). This result indicates a strong impact of heterogeneity across cohorts because *p*_*Fisher*_ in this case was typically smaller than *p*_*meta*_ for different phenotypes in pathway 1a (Supplementary Tables 4C,D).

Pathway 2 provides a natural opportunity to validate the antagonistic heterogeneity for 81 SNPs in different cohorts. Our analysis shows that this heterogeneity, characterized by *p*_*OpFc*_ < *p*_*FpFc*_ with a difference of ≥0.2 orders of magnitude, was replicated for all SNPs. For 5 SNPs this difference was observed in two cohorts and for the remaining 76 SNPs in three to seven cohorts (Supplementary Table 8).

### Biological Pathway and Gene Ontology (GO) Enrichment Analysis

We performed enrichment analyses of biological pathways and toxicity (tox) functions using IPA (www.qiagenbioinformatics.com) and GO biological processes (BPs) using DAVID (Huang da et al., 2009) in two groups of four and two gene sets. The first group included four gene sets: (i) three sets for pleiotropic SNPs from Table 3 (pw1), Table 4 (pw2), and the two tables combined (pw1&2) and (ii) one set for the AlbU-specific SNPs from Table 2. The second group included genes for SNPs from the identified conventional and unconventional sets. The conventional set of 59 SNPs (Tables 3, 4, groups D, C, CB, and M) characterizes associations commonly expected in genetic association studies. The unconventional set of 107 SNPs is characterized by strong impact of antagonistic heterogeneity (Tables 3, 4, groups HP and HB).

**Figure 5:**
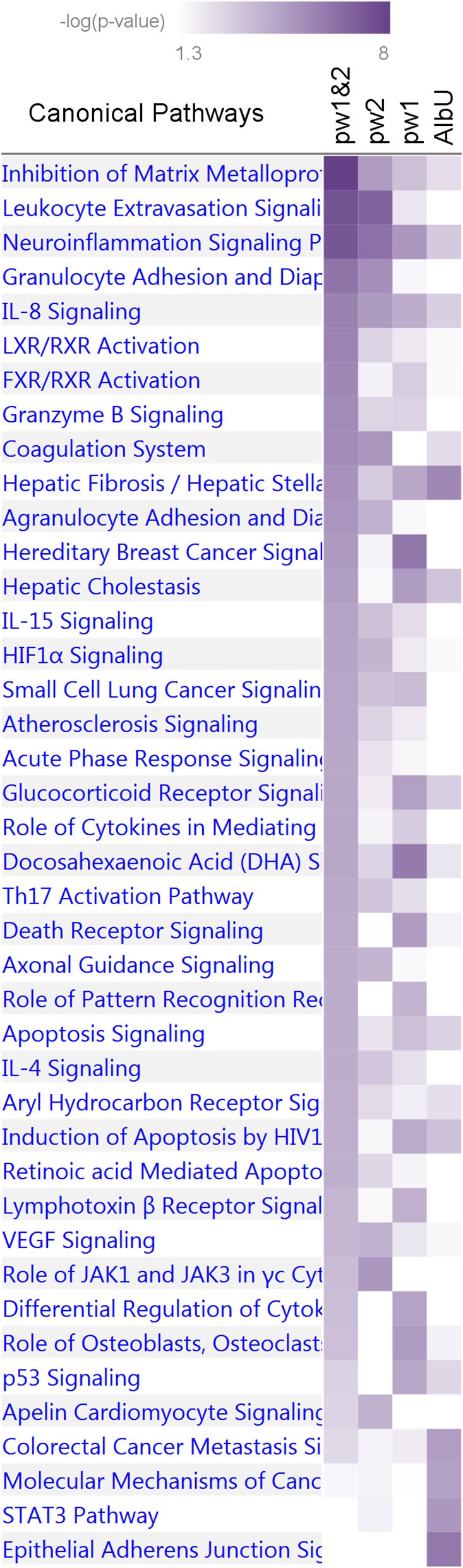
Enrichment of canonical biological pathways in four gene sets. Pw1, pw2, and pw1&2 denote three sets of genes for pleiotropic SNPs from Tables 3, 4 separately and combined, respectively. AlbU denotes the set of genes for SNPs associated with albumin in urine (AlbU) from Table 2. Color coding for –log_10_ (*p*-value) is given in the inset. Numerical estimates are given in Supplementary Table 9.

The IPA analysis of the first group of four sets identified 41 pathways (Figure 5 and Supplementary Table 9) enriched for genes in at least one set at *p* < 10^−4^ (Fisher's exact test). Five pathways, *inhibition of matrix metalloproteases, neuroinflammation signaling, hepatic fibrosis/hepatic stellate cell activation, IL-8 signaling*, and *apoptosis signaling*, were consistently enriched for pleiotropic genes from the pw1 and pw2 sets (*p* < 10^−2^), with stronger enrichment attained in the combined pw1&2 gene set (*p* < 10^−4^) as well as for genes from the AlbU set (*p* < 10^−2^). One pathway, *docosahexaenoic acid (DHA) signaling*, also attained the same cut offs for the significance, although *p*-value in the combined pw1&2 set was larger than that in the pw1 set. Pooling genes from the pw1 and pw2 sets into the pw1&2 set improved the significance of the enrichment for most biological pathways (25 of 41) in the pw1&2 set. One pathway, *differential regulation of cytokine production in macrophages and T helper cells by IL-17A and IL-17F*, was enriched (*p* < 10^−4^) exclusively in the pw1 set with no genes in the pw2 set. Of 30 pathways enriched in the pw1&2 set at *p* < 10^−4^, 15 pathways were either not present (no genes) or did not attain nominal significance (*p* > 0.05) in the AlbU set.

**Figure 6:**
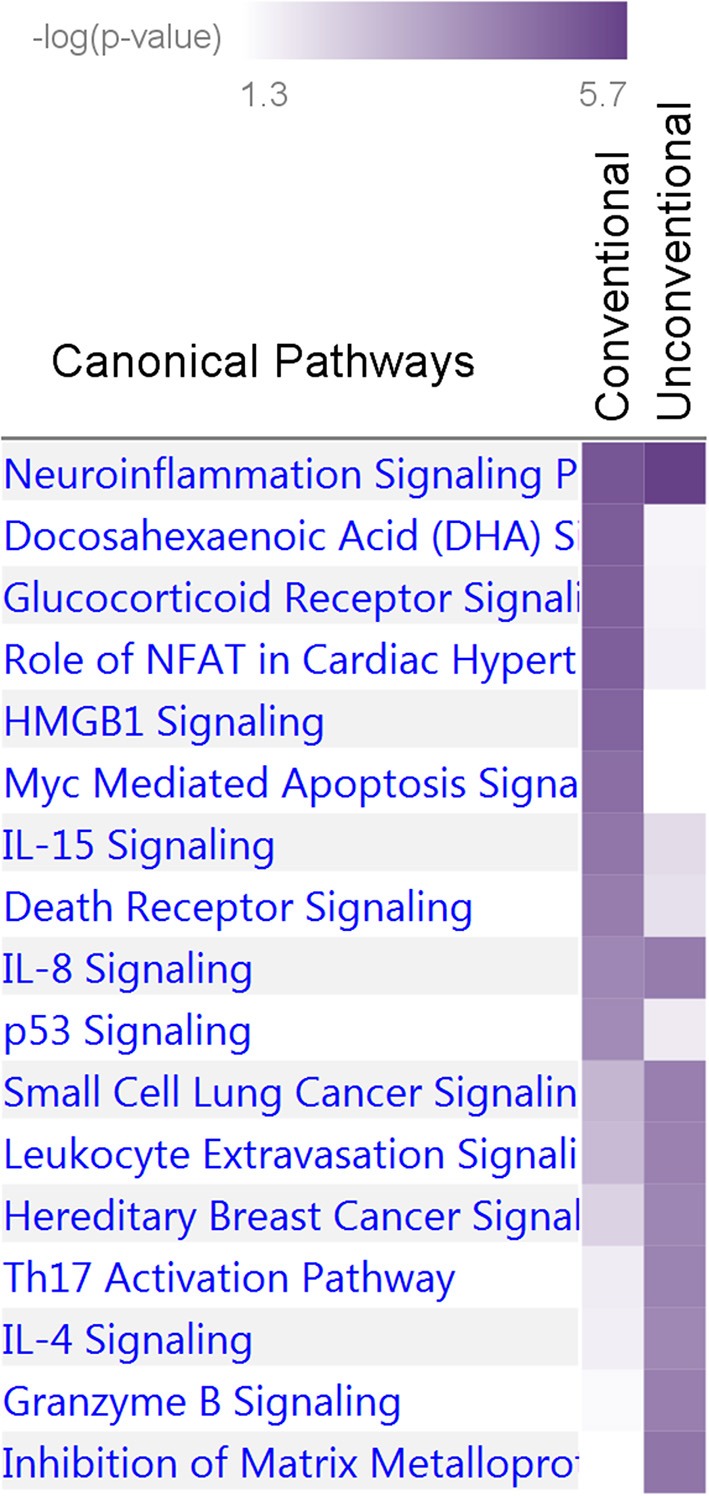
Enrichment of canonical biological pathways in two gene sets. Conventional set includes genes for pleiotropic SNPs from Tables 3, 4, groups D, C, CB, and M. Unconventional set includes genes for pleiotropic SNPs from Tables 3, 4, groups HB and HP. The conventional set characterizes associations commonly expected in genetic association studies whereas the unconventional set highlights a phenomenon characterized by strong impact of antagonistic heterogeneity. Color coding for –log_10_ (*p*-value) is given in the inset. Numerical estimates are given in Supplementary Table 10.

Nine and 10 pathways were enriched at *p* < 10^−4^ in the unconventional and conventional sets, respectively (Supplementary Table 10). Two of them, *neuroinflammation signaling* and *IL-8 signaling* pathways, attained *p* < 10^−4^ in both sets whereas the others predominantly characterized the unconventional or conventional set. Thus, this analysis identified 17 pathways enriched at *p* < 10^−4^ in the two sets (Figure 6). Fourteen of these Seventeen pathways were enriched at *p* < 10^−4^ in the pw1, pw2, and/or pw1&2 gene sets (Supplementary Table 10). Analysis of the tox functions for these two sets identified 43 terms enriched at *p* < 10^−4^ in the conventional (20 terms) and unconventional (27 terms) sets with four terms related to cardiotoxicity attaining *p* < 10^−4^ in both sets (*enlargement of heart, hypertrophy of heart, arrhythmia*, and *familial arrhythmia*) (Supplementary Table 11). Concurring with the pathway analysis, these terms supported differences in tox functions in the unconventional and conventional sets.

The GO analysis identified 8 BPs with enrichment for genes at *p* < 10^−4^ (Fisher's exact test) in at least one of the four sets, i.e., pw1, pw2, pw1&2, and unconventional sets; no enrichment at *p* < 10^−4^ was observed in AlbU and conventional sets (Supplementary Table 12). These BPs were associated with immune and inflammatory process (e.g., *positive regulation of B cell proliferation, platelet degranulation, response to drug, response to hypoxia*), extracellular matrix (*extracellular matrix disassembly*), the coagulation system (*platelet degranulation, response to hypoxia)*, and specific metabolic events (*triglyceride metabolic process*). BP *positive regulation of B cell proliferation* showed the most significant enrichment (*p* = 9.3 × 10^−7^) in the pw1&2 set and it was the only pathway enriched at *p* < 0.01 in all five pleiotropic sets (i.e., excluding AlbU).

## Discussion

Despite a modest sample of 26,371 individuals from five longitudinal studies, our comprehensive univariate and pleiotropic meta-analyses identified large number of 237 novel SNPs in 199 loci with phenotype-specific (25.7%) and pleiotropic (74.3%) associations and replicated associations for 160 SNPs in 68 loci. We show that most previously reported SNPs, which were replicated in our univariate meta-analysis (98.1%, 157 of 160 SNPs), exhibited relatively homogeneous associations in the studied cohorts. Consistent with the framework of medical genetics, this result provides evidence that currently prevailing strategies of large-scale GWAS are well adapted to handle homogeneous genetic effects. This result is contrasted by a substantial impact of heterogeneity, with the natural-selection—free genetic heterogeneity as its inevitable component, in a large fraction of the 237 novel SNPs identified in our univariate (18 of 61 SNPs, 29.5%) and pleiotropic (115 of 176 SNPs, 65.3%) meta-analyses. In pleiotropic meta-analysis, this result underscores heterogeneous pleiotropy and its most common type, antagonistic genetic heterogeneity (107 of 115 SNPs, 93%), which is characterized by antagonistic directions of genetic effects for directly correlated phenotypes (Figures 2D, 4). Although antagonistic genetic heterogeneity is counter-intuitive in medical genetics, it is naturally expected when molecular mechanisms of age-related phenotypes are not due to direct evolutionary selection. This heterogeneity also has been reported for alleles from well-known apolipoprotein B gene (Kulminski et al., 2017), which are involved in lipid metabolism. It provides unprecedented insight into the genetic origins of age-related phenotypes and side effects of medical care. Unlike the common role of antagonistic genetic heterogeneity in pleiotropic associations, our results do not support a common role of mediated pleiotropy (Solovieff et al., 2013) (15 of 176 SNPs, 8.5%), which is conventionally assumed for correlated phenotypes, especially in framework of medical genetics (Teslovich et al., 2010).

Most pleiotropic SNPs (137 of 176, 77.8%) attained GW significance in the two largest domains of 16 quantitative markers (60.2%) and 24 phenotypes (17.6%), which supports the hypothesis that genetic associations with multiple age-related phenotypes can be driven by more fundamental mechanisms than those underlying common etiologies of phenotypes (Goh et al., 2007; Franco et al., 2009; Kirkwood et al., 2011; Kulminski et al., 2016a). This idea has been conceptualized in the rapidly developing discipline of geroscience (Kaeberlein et al., 2015; Franceschi and Garagnani, 2016), which is based on observations that age and aging (Guarente, 2011) are among the most important risk factors for geriatric diseases of distinct etiologies. This pleiotropy may provide a basis for improving health care by reducing the burden of a major subset of common diseases (Martin et al., 2007; Sierra et al., 2008). However, the dominant role of antagonistic heterogeneity in pleiotropic associations cautions against simplistic approaches in studies of the genetic origin of multiple age-related phenotypes. Antagonistic heterogeneity emphasizes the importance of personalized medicine (Schork, 2015), which can potentially handle antagonistic risks [due to gene-gene and/or gene-environment interactions (Jazwinski et al., 2010; Ukraintseva et al., 2016)] on an individual basis. Our results suggest that merging geroscience and personalized medicine is highly promising for the efficient translation of discoveries regarding the genetics of age-related phenotypes to health care developments.

The degree of similarity or mismatch of correlation patterns between genetic and phenotypic factors is of enduring interest in the evolutionary genetics as it informs about forces driving development and clustering of phenotypes (Cheverud, 1988). For example, the similarity of the patterns suggests that the same environmental exposures contribute to the genetic and phenotypic variation (Cheverud, 1988) whereas their mismatch indicates that different exposures likely affect them (Hebert et al., 1994). If a study considers phenotypes directly shaped by natural selection (e.g., fitness-related phenotypes), the analysis of the degree of similarity or mismatch leveraging the genetic basis of pleiotropy helps in dissecting pathways leading to organisms' morphological modularity (Cheverud et al., 2004). As the age-related phenotypes are considered not to be directly shaped by natural selection (Nesse and Williams, 1994; Nesse et al., 2012), it is unlikely that this analysis of such phenotypes can help in gaining insights into organisms' morphology. Still, it informs on potential role of the environment. Specifically, co-direction of the genetic associations with phenotypes and correlation between them may indicate the mechanism of biological or mediated pleiotropy (Solovieff et al., 2013) shaped by the same environment. Anti-collinearity of the genetic associations and correlation between phenotypes likely indicates heterogeneity of genetic mechanisms in pleiotropic predisposition to such phenotypes shaped by different environmental exposures. Thus, our finding of extensive effect of antagonistic heterogeneity driven by such anti-collinearity supports a key role of the environment in shaping heterogeneous pleiotropic predisposition to age-related phenotypes.

Our bioinformatics analysis of enrichment of canonical pathways in two groups of four (pw1, pw2, pw1&2, and AlbU) and two (conventional and unconventional) gene sets identified 41 and 17 pathways at *p* < 10^−4^. The signature of these two groups was enrichment in the immune/inflammation responses, 14 of 41 (Supplementary Table 9) and 8 of 17 (Supplementary Table 10) pathways, respectively. Of them, two pathways (*neuroinflammation signaling* and *IL-8 signaling*) were enriched in all six sets and four (*inhibition of matrix metalloproteases, hepatic fibrosis, apoptosis signaling*, and *docosahexaenoic acid (DHA) signaling*) in four sets of the first group (pw1, pw2, pw1&2, and AlbU). Neuroinflammation, an inflammatory response within the central nervous system, is closely associated with neurodegenerative diseases such as the Alzheimer's and Parkinson's diseases (Fulop et al., 2018). Pro-inflammatory cytokine *IL-8* plays an important role in pathological aging as it promotes angiogenesis and tumorigenicity (Qazi et al., 2011); it was also linked to cardiovascular disease and neuroinflammation (Apostolakis et al., 2009; Ramesh et al., 2013). Matrix metalloproteases (MMPs), which are extracellular matrix-degrading enzymes, are also involved in the modulation of inflammation and the innate immune system (Sorokin, 2010). MMPs are associated with the development of various age-related diseases including cancer, cardiovascular pathologies, neurological diseases, inflammatory, and fibrotic disorders (Freitas-Rodriguez et al., 2017). Hepatic fibrosis is associated with the expansion of potential cellular sources of extracellular matrix and crosstalk with inflammatory and immune systems (Jiao et al., 2009). Apoptosis occurs naturally as a mechanism of maintaining cell populations in tissues and as a defense against harmful stimuli and pathological processes via the immune response (Strasser et al., 2000). DHA is relevant for brain function especially in the context of neuroinflammation (Hashimoto et al., 2017). The analysis of top GO BPs supported enrichment of pathways related to inflammation and immunity. Specifically, B cells *(positive regulation of B cell proliferation)* play an important role in regulation of immune responses and inflammation (Bouaziz et al., 2008; Cain et al., 2009). BP *extracellular matrix disassembly*, tightly linked with MMPs, has a role in physiological and pathological traits (e.g., tissue fibrosis, cancer, cardiovascular disease, arthritis) and in mediating immune responses and tissue inflammation (Sorokin, 2010; Bonnans et al., 2014).

Comparative analysis of enrichment of pathways in the conventional and unconventional sets identified death and stress response as characteristic processes for the conventional set and maintenance of extracellular matrix (ECM) components and tissue homeostasis as those for the unconventional set. The death/stress response is supported by enrichment of genes in three apoptotic signaling pathways, *myc mediated apoptosis signaling, death receptor signaling, IL-15 signaling* (Figure 6, Supplementary Table 10). In addition, *HMGB1* (*HMGB1 signaling*) codes a proinflammatory cytokine, which is critical to the cell response to stress and is implicated in diseases characterized by cell damages and death (e.g., Alzheimer's disease, stroke, cancer) (Bell et al., 2006). As a response to stress, *p53 signaling* can activate multiple apoptotic signals (Vousden and Lane, 2007). Glucocorticoids (*glucocorticoid receptor signaling*) are essential for stress response (Oakley and Cidlowski, 2013). The unconventional-set–specific role of ECM/tissue homeostasis is supported by *inhibition of matrix metalloproteases, granzyme B signaling, IL-4 signaling*, and *Th17 activation* pathways. The extracellular proteases, such as the MMPs, are a regulator of extracellular matrix turnover playing a role in tissue development, repair and remodeling (Stamenkovic, 2003). The serine protease granzyme B is implicated in degradation of extracellular matrix proteins, particularly, influencing age-specific impaired wound healing (Hiebert and Granville, 2012; Parkinson et al., 2015). The pleiotropic cytokine IL-4 may promote biogenesis of extracellular matrix proteins in normal wound healing and in pathological fibrosis (Postlethwaite et al., 1992; Salmon-Ehr et al., 2000). The IL-17, the key cytokine produced by Th17 cells, contributes to tissue inflammation associated with extracellular matrix destruction and activates the production and function of matrix metalloproteinases (Miossec and Kolls, 2012). In line, death and cardiotoxicity terms characterize tox functions in the conventional set whereas hepatotoxicity, which includes liver cancers tightly linked with tissue homeostasis, along with cardiotoxicity are characteristic tox functions for the unconventional set.

Thus, the results of our bioinformatics analysis suggest that inflammation and immune responses, which are critical processes in aging (Finch et al., 2010), may play a key role in genetic predisposition to multiple phenotypes, highlighting inflammation and immune responses as promising targets in geroscience and personalized medicine (Franceschi et al., 2018). They also provide first insights into potential biological functions related to tissue homeostasis, which may underline the antagonistic genetic heterogeneity.

In summary, our analysis shows that genetic association studies relying on the rigor of large samples are adapted to handle homogeneous genetic effects. Most genetic associations with complex, age-related traits examined in this study are, however, inherently heterogeneous. Accordingly, large fraction of variance in genetic predisposition to such traits may be missed within the traditional framework. Leveraging more comprehensive analyses adapted to deal with the inherent heterogeneity in genetic predisposition to age-related traits is critical to substantially advance the progress in uncovering genetic architecture of such traits.

## Author Contributions

AK conceived and designed the experiment and wrote the paper. YL performed statistical analyses and contributed to drafting of the paper. JH, KA, OB, and SU prepared data. AY contributed to discussion of the intermediate and final results. IC prepared data and performed bioinformatics analysis.

### Conflict of Interest Statement

The authors declare that the research was conducted in the absence of any commercial or financial relationships that could be construed as a potential conflict of interest.
