# Supplementary material for: Pleiotropic Meta-Analysis of Age-Related Phenotypes Addressing Evolutionary Uncertainty in Their Molecular Mechanisms
Source: Front Genet. 2019 May 10;10:433. doi: 10.3389/fgene.2019.00433 (PMC6524409; doi:10.3389/fgene.2019.00433)
Supplement: Supplementary file 12 [file Data_Sheet_1.docx]

Supplementary Material

# Supplementary Note

## Acknowledgment Text

This research was supported by Grants No P01 AG043352 and R01AG047310 from the National Institute on Aging. The funders had no role in study design, data collection and analysis, decision to publish, or preparation of the manuscript. The content is solely the responsibility of the authors and does not necessarily represent the official views of the National Institutes of Health. This manuscript was prepared using a limited access datasets obtained though dbGaP (accession numbers phs000007.v28.p10, phs000280.v3.p1, phs000209.v13.p3, phs000287.v5.p1, phs000285.v3.p2).

The Framingham Heart Study (FHS) is conducted and supported by the National Heart, Lung, and Blood Institute (NHLBI) in collaboration with Boston University (Contract No. N01-HC-25195 and HHSN268201500001I). This manuscript was not prepared in collaboration with investigators of the FHS and does not necessarily reflect the opinions or views of the FHS, Boston University, or NHLBI. Funding for CARe genotyping was provided by NHLBI Contract N01-HC-65226. Funding support for the Framingham Dementia dataset was provided by NIH/NIA grant R01 AG08122. Funding support for the Framingham Inflammatory Markers was provided by NIH grants R01 HL064753, R01 HL076784 and R01 AG028321. Funding support for the Framingham C-reactive Protein dataset was provided by NIH grants R01 HL064753, R01 HL076784 and R01 AG028321. Funding support for the Framingham Adiponectin dataset was provided by NIH/NHLBI grant R01-DK-080739. Funding support for the Framingham Interleukin-6 dataset was provided by NIH grants R01 HL064753, R01 HL076784 and R01 AG028321.

The Cardiovascular Health Study (CHS) was supported by contracts HHSN268201200036C, HHSN268200800007C, N01-HC-85079, N01-HC-85080, N01-HC-85081, N01-HC-85082, N01-HC-85083, N01-HC-85084, N01-HC-85085, N01-HC-85086, N01-HC-35129, N01 HC-15103, N01 HC-55222, N01-HC-75150, N01-HC-45133, and N01-HC-85239; grant numbers U01 HL080295 and U01 HL130014 from the National Heart, Lung, and Blood Institute (NHLBI), and R01 AG-023629 from the National Institute on Aging, with additional contribution from the National Institute of Neurological Disorders and Stroke. A full list of principal CHS investigators and institutions can be found at https://chs-nhlbi.org/pi. This manuscript was not prepared in collaboration with CHS investigators and does not necessarily reflect the opinions or views of CHS, or the NHLBI. Support for the genotyping through the CARe Study was provided by NHLBI Contract N01-HC-65226.

The Atherosclerosis Risk in Communities Study (ARIC) is carried out as a collaborative study supported by National Heart, Lung, and Blood Institute contracts (HHSN268201100005C, HHSN268201100006C, HHSN268201100007C, HHSN268201100008C, HHSN268201100009C, HHSN268201100010C, HHSN268201100011C, and HHSN268201100012C). The authors thank the staff and participants of the ARIC study for their important contributions. Funding for CARe genotyping was provided by NHLBI Contract N01-HC-65226.

The Coronary Artery Risk Development in Young Adults Study (CARDIA) is conducted and supported by the National Heart, Lung, and Blood Institute (NHLBI) in collaboration with the University of Alabama at Birmingham (N01-HC95095 & N01-HC48047), University of Minnesota (N01-HC48048), Northwestern University (N01-HC48049), and Kaiser Foundation Research Institute (N01-HC48050). This manuscript was not approved by CARDIA. The opinions and conclusions contained in this publication are solely those of the authors, and are not endorsed by CARDIA or the NHLBI and should not be assumed to reflect the opinions or conclusions of either. Funding for CARe genotyping was provided by NHLBI Contract N01-HC-65226.

MESA and the MESA SHARe project are conducted and supported by the National Heart, Lung, and Blood Institute (NHLBI) in collaboration with MESA investigators. Support for MESA is provided by contracts N01-HC- 95159, N01-HC-95160, N01-HC-95161, N01-HC-95162, N01-HC-95163, N01-HC-95164, N01-HC-95165, N01-HC- 95166, N01-HC-95167, N01-HC-95168, N01-HC-95169, UL1-RR-025005, and UL1-TR-000040. This manuscript was not prepared in collaboration with MESA investigators and does not necessarily reflect the opinions or views of MESA, or the NHLBI. Funding for CARe genotyping was provided by NHLBI Contract N01-HC-65226. Funding support for the adiponectin dataset was provided by grant 1R01HL088451-01A1.

# Supplementary Figures and Tables

## Supplementary Figures


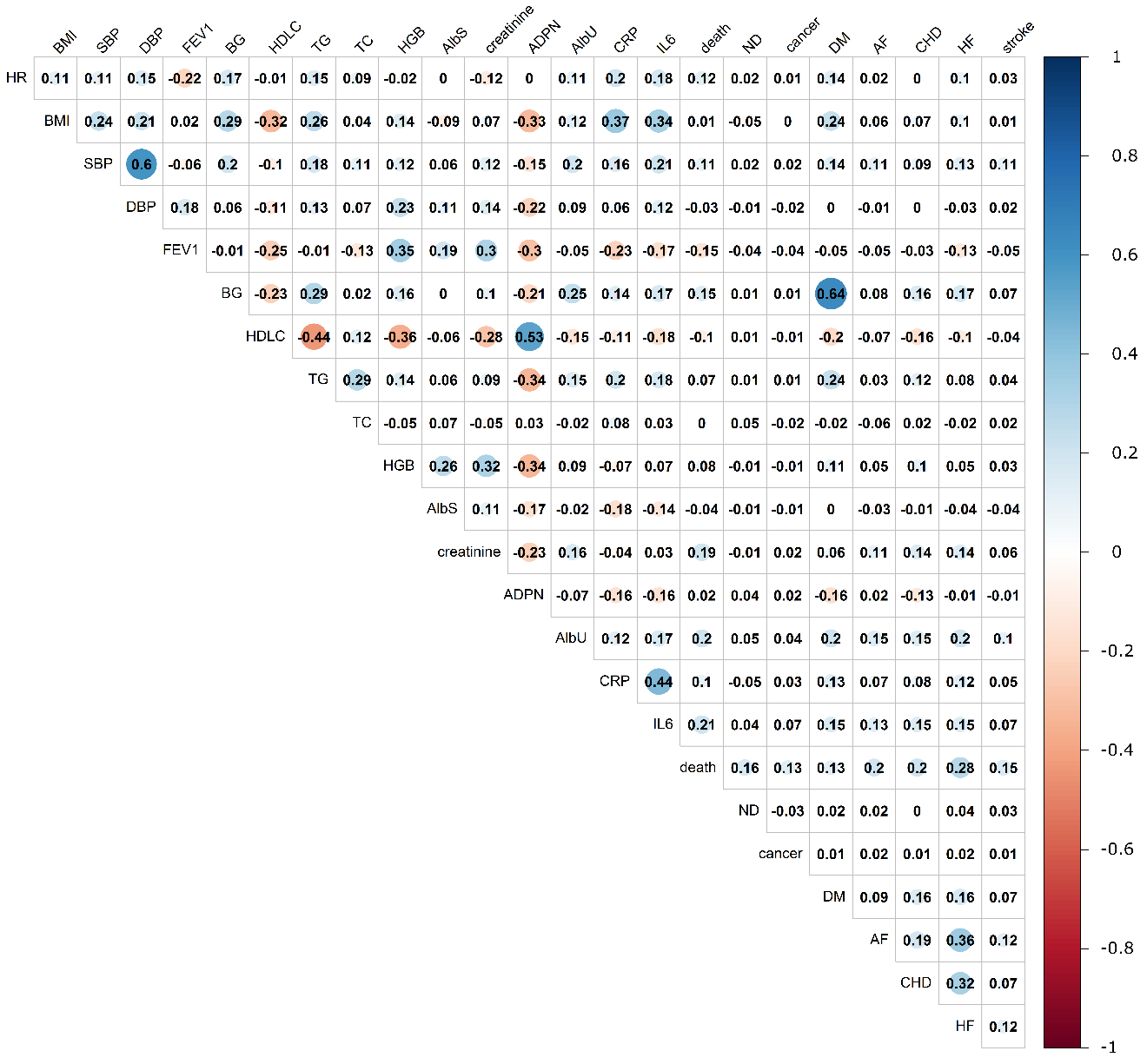


**Supplementary Figure 1.** **Pairwise correlation coefficients** $\Sigma_{m}^{P}$**between phenotypes selected for the analysis.** Correlation coefficients were taken from matrix $\boldsymbol{\Sigma}_{\boldsymbol{m}}^{\boldsymbol{P}}$ constructed using a fixed effect meta-test applied to the correlation statistics of phenotypes in each cohort (see Methods). Phenotypes include: adiponectin (ADPN); albumin in urine (AlbU); albumin serum (AlbS); blood glucose (BG); body mass index (BMI); C-reactive protein (CRP); creatinine; diastolic blood pressure (DBP); forced expiratory volume in 1 second (FEV1); heart rate (HR); hemoglobin (HGB); high-density lipoprotein cholesterol (HDLC); interleukin 6 (IL6); systolic blood pressure (SBP); total cholesterol (TC); and triglycerides (TG); and information on events for 7 diseases: atrial fibrillation (AF); cancer; coronary heart disease (CHD); diabetes mellitus (DM); heart failure (HF); dementia of Alzheimer type (ND), stroke, and death.


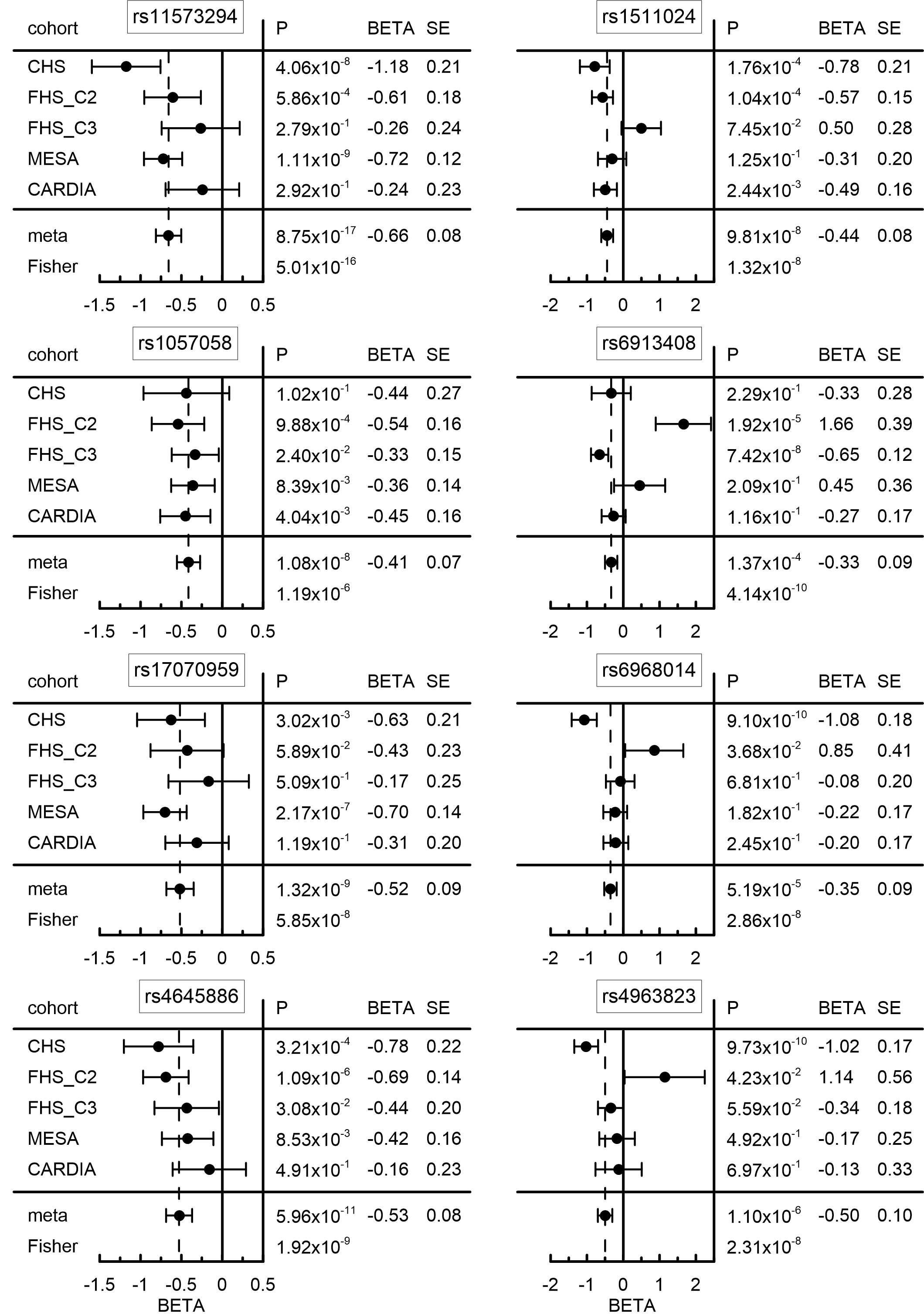


**Supplementary Figure 2. Forest plots illustrating homogeneous and heterogeneous associations.** (Left panel) homogeneous and (right panel) heterogeneous associations across cohorts for eight uncommon SNPs associated with albumin in urine. “Meta” denotes the results of the fixed-effect meta-test and “Fisher” denotes the results of the Fisher test in five cohorts: the Cardiovascular Health Study (CHS), the Coronary Artery Risk Development in Young Adults (CARDIA) study, the Multi-Ethnic Study of Atherosclerosis (MESA), the Framingham Heart Study Offspring (FHS_C2) and the 3^rd^ generation (FHS_C3) cohorts. SE denotes standard error. Bars show 95% confidence intervals. BETA, SE and P denote effect size, standard error and *p*-value, respectively.


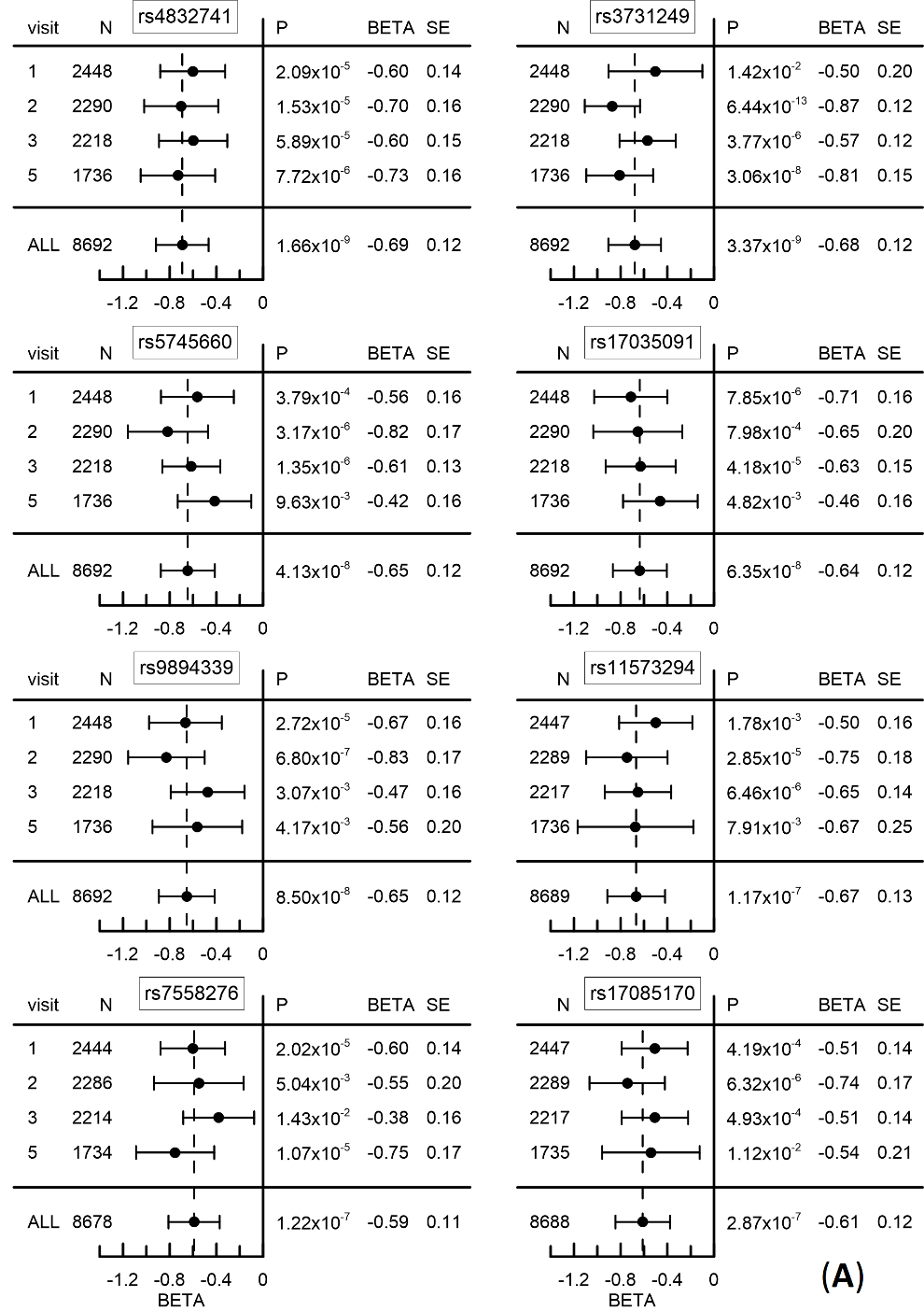


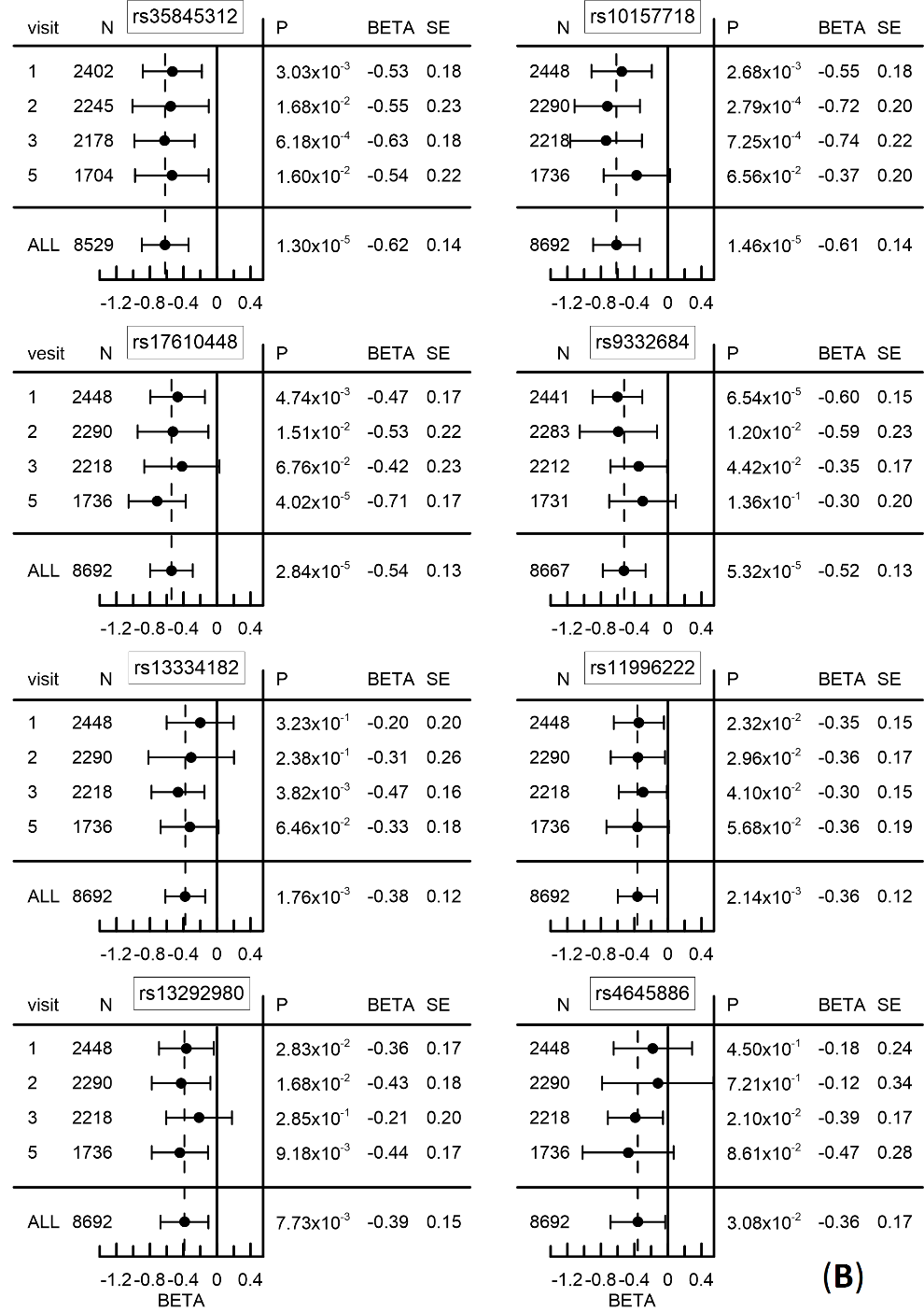


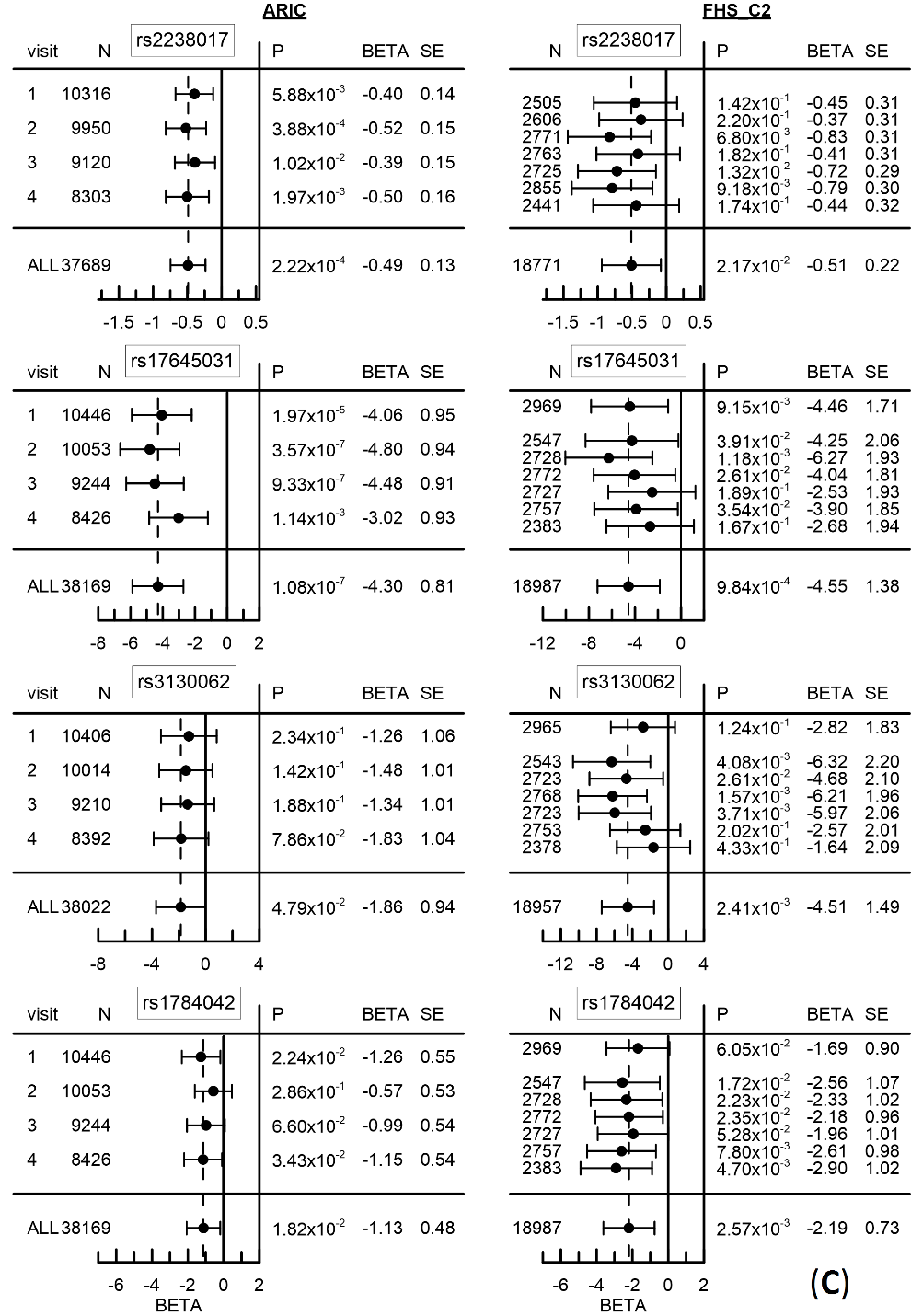


**Supplementary Figure 3. Forest plots illustrating benefits of longitudinal information.** (A and B) 16 SNPs associated with albumin in urine at four visits available in MESA (1, 2, 3, and 5). (C) Four SNPs associated with total cholesterol in the Atherosclerosis Risk in Communities (ARIC) study (left) and the Framingham Heart Study Offspring (FHS_C2) cohort (right). N indicates the sample size at a given visit and person-observations. “All” denotes the estimates evaluated leveraging longitudinal information. SE denotes standard error. Bars show 95% confidence intervals. BETA, SE and P denote effect size, standard error and *p*-value, respectively.


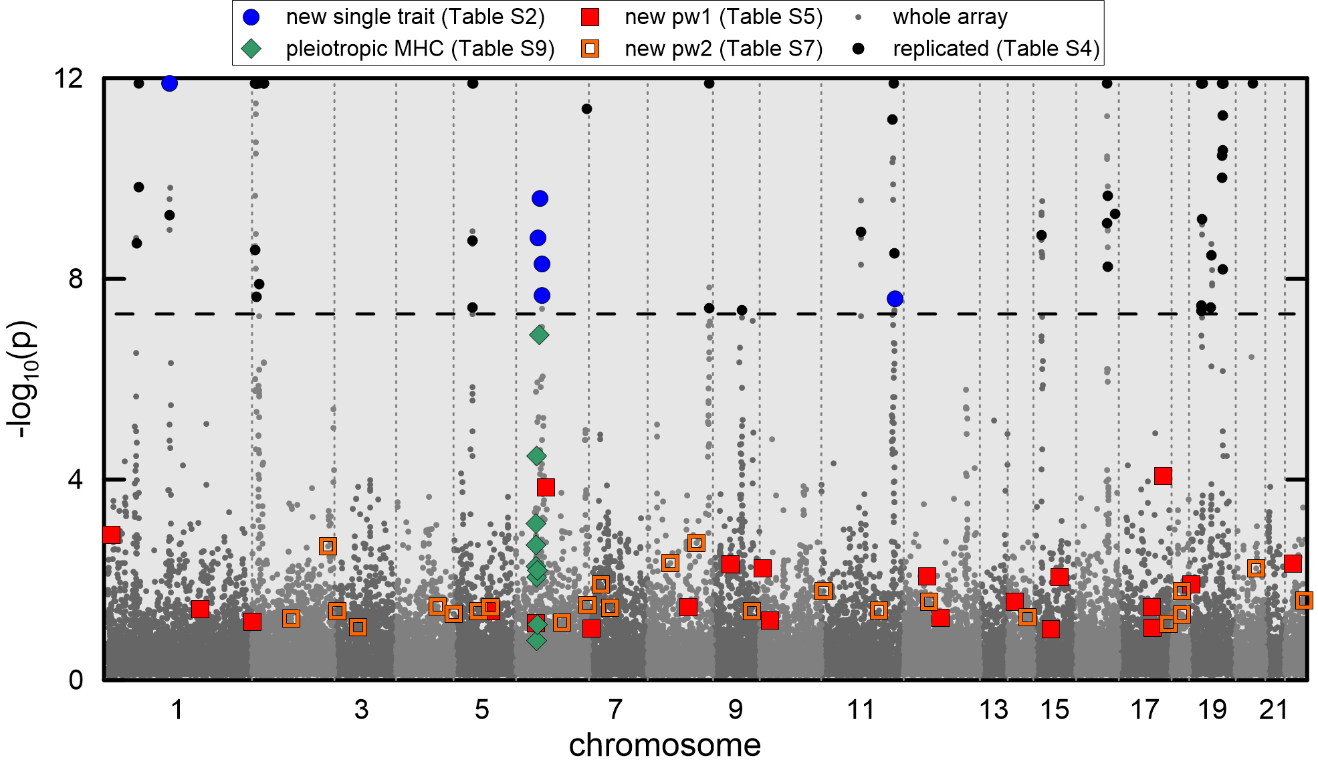


**Supplementary Figure 4. Manhattan plot for total cholesterol.** Dashed horizontal line shows GW level -log_10_(5×10^-8^)=7.3. Larger symbols show novel SNPs and SNPs from the MHC. The data is for SNPs from the univariate meta-analysis from Table 2 (blue dots) and pleiotropic meta-analyses from Supplementary Table 6 (green diamonds), Table 3 (filled red squares), and Table 4 (open red squares). Replicated SNPs from Supplementary Table 3 are shown by smaller black dots. Small gray dots show the results of univariate meta-analysis of the other SNPs on the array.

## Supplementary Tables

**Supplementary Table 1.** Basic characteristics of cohorts included in the analyses and available sample sizes.

| **Variable** | **ARIC** | **CHS** | **FHS_C1** | **FHS_C2** | **FHS_C3** | **MESA** | **CARDIA** |
| --- | --- | --- | --- | --- | --- | --- | --- |
| Sample size | 10,540 | 4,174 | 639 | 3,062 | 3,960 | 2,474 | 1,522 |
| Age (SD), years | 54.3 (5.7) | 72.8 (5.6) | 35.8 (4.3) | 34.8 (9.8) | 40.2 (8.8) | 62.7 (10.3) | 25.6 (3.4) |
| BC range, years | 1920-1945 | 1885-1925 | 1895-1920 | 1910-1965 | 1930-1980 | 1917-1957 | 1950-1965 |
| Gender, female (%) | 5,617 (53.3) | 2,355 (56.4) | 428 (67.0) | 1,655 (54.1) | 2,104 (53.1) | 1,292 (52.2) | 809 (53.2) |
| Quantitative markers: number of visits (number of person-visits available for the analyses) | | | | | | | |
| ADPN, [ng/ml] | N/A | 1 (992) | N/A | 1 (2,313) | 1 (3,943) | 1 (744) | N/A |
| AlbU [mg/dl] | N/A | 1 (2,432) | N/A | 1 (2,364) | 1 (3,289) | 4 (8,742) | 2 (2,598) |
| AlbS [g/dl] | 1 (10,468) | 3 (9,918) | 1 (440) | 2 (5,355) | 1 (3,289) | N/A | 1 (1,515) |
| BG [mg/dl] | 4 (38,196) | 3 (9,918) | 22 (10,896) | 6 (16,004) | 1 (3,951) | 5 (10,863) | 4 (5,704) |
| BMI [kg/m^2^] | 4 (38,162) | 3 (9,986) | 23 (10,940) | 8 (21,596) | 1 (3,948) | 5 (10,885) | 6 (8,580) |
| CRP [mg/l] | 2 (8,813) | 2 (7,486) | N/A | 3 (7,796) | 2 (7,233) | 2 (2,661) | 2 (2,795) |
| Creatinine [mg/dl] | 3 (28,959) | 3 (10,437) | 8 (3,138) | 4 (10,217) | 2 (7,233) | 4 (8,567) | 3 (4,321) |
| DBP [mmHg] | 4 (38,222 ) | 9 (28,700) | 28 (15,773) | 8 (21,960) | 1 (3,953) | 5 (10,916) | 6 (8,695) |
| FEV1 [liter] | 2 (20,313) | 3 (9,102) | 5 (2,537) | 5 (10,573) | 1 (3,709) | 2 (1,302) | 4 (5,740) |
| HR [beat/min] | 4 (38,070) | 10 (31,534) | 26 (13,953) | 7 (18,921) | 1 (3,953) | 2 (4,172) | 4 (3,225) |
| HGB [g/dl] | 4 (28,950) | 2 (7,868) | 7 (3,640) | 2 (5,167) | 1 (2,379) | N/A | 1 (1,515) |
| HDL-C [mg/dl] | 4 (38,135) | 2 (7,537) | 11 (4,694) | 8 (21,430) | 1 (3,949) | 5 (10,849) | 6 (8,613) |
| IL6 [pg/ml] | 1 (336) | N/A | N/A | 1 (2,756) | 1 (3,910) | 1 (728) | N/A |
| SBP [mmHg] | 4 (38,222) | 9 (28, 764) | 28 (15,779) | 8 (21,962) | 1 (3,953) | 5 (10,916) | 6 (8,696) |
| TC [mg/dl] | 4 (38,169) | 8 (21,073) | 22 (10,643) | 7 (19,001) | 2 (7,245) | 5 (10,856) | 6 (8,614) |
| TG [mg/dl] | 4 (38,186) | 2 (7,548) | 9 (2,789) | 8 (21,450) | 1 (3,951) | 5 (10,860) | 6 (8,615) |
| Risk outcomes: number of cases available for the analyses | | | | | | | |
| Follow up through | 2004 | 2004 | 2012 | 2012 | 2012 | 2012 | 2001 |
| AF | 1,021 | 1,089 | 239 | 424 | N/A | 132 | N/A |
| CHD | 966 | 1,297 | 223 | 469 | N/A | 163 | N/A |
| HF | 889 | 1,162 | 187 | 218 | N/A | 71 | N/A |
| Stroke | 396 | 421 | 116 | 171 | N/A | 56 | N/A |
| DM | 836 | 693 | 196 | 287 | N/A | 376 | 107 |
| ND | NA | 242 | 205 | 103 | N/A | N/A | N/A |
| Cancer | 1,422^*^ | 834 | 198 | 655 | 161 | 313^*^ | N/A |
| Death | 1,536 | 2,125 | 527 | 489 | N/A | 176 | N/A |

^*^ All cancer sites combined; otherwise, all cancer sites except skin.

**Cohorts**: Atherosclerosis Risk in Communities Study (ARIC); Cardiovascular Health Study (CHS); Framingham Heart Study (FHS) original cohort (FHS_C1); FHS Offspring (FHS_C2); FHS 3^rd^ generation cohort (FHS_C3), the Multi-Ethnic Study of Atherosclerosis (MESA), and Coronary Artery Risk Development in Young Adults (CARDIA).

**Quantitative markers**: Adiponectin (ADPN); Albumin in urine (AlbU); Albumin in serum (AlbS); blood glucose (BG); body mass index (BMI); C-reactive protein (CRP); creatinine; diastolic blood pressure (DBP); forced expiratory volume in 1 second (FEV1); heart rate (HR); hemoglobin (HGB); high-density lipoprotein cholesterol (HDL-C); interleukin 6 (IL6); systolic blood pressure (SBP); total cholesterol (TC); triglycerides (TG).

**Risk outcomes**: atrial fibrillation (AF), coronary heart disease (CHD), heart failure (HF), stroke, diabetes mellitus (DM), dementias of Alzheimer’s type (ND), cancer, and death.

**N/A** identifies phenotypes not available in a given cohort.

BC denotes birth cohorts; SD denotes standard deviation.

**Supplementary Table 2.** Supplemental information for Table 2. (A) Linkage disequilibrium (LD, *r^2^*) for SNPs from the univariate meta-analysis of individual phenotypes reported in Table 2, which are within ±1Mb flanking region. LD was evaluated in MESA with cut off *r^2^*=0.01. (B) Cohort-specific results for SNPs from the univariate meta-analysis of individual phenotypes reported in Table 2.

See Supplementary File Table_S02.xlsx

**Supplementary Table 3.** SNPs replicated in the univariate meta-analysis of individual phenotypes. (A). Linkage disequilibrium (LD, *r^2^*) for replicated SNPs reported in Supplementary Table 3B, which are within ±1Mb flanking region. LD for most SNPs was evaluated in ARIC. For SNPs missing in ARIC, we used information on SNPs available in: CARDIA, FHS, CHS and MESA. Cut off for LD was *r^2^*=0.01. (B) Replicated SNPs attained genome-wide significance (*p* < 5×10^‑8^) in the univariate meta-analysis of individual phenotypes.

See Supplementary File Table_S03.xlsx

**Supplementary Table 4.** Supplemental information for Table 3. (A) Linkage disequilibrium (LD, *r^2^*) for SNPs from the pleiotropic meta-analysis in pathway 1 reported in Table 3, which are within ±1Mb flanking region. LD was evaluated in ARIC with cut off *r^2^*=0.01. (B) The results from the fixed-effect meta-analysis of the associations of SNPs with each of 24 phenotypes used to create Table 3 (pathway 1a). (C) The results from the Fisher meta-analysis of the associations of SNPs with each of 24 phenotypes used to create Table 3 (pathway 1a). (D) The results from four pleiotropic meta-analyses for SNPs attained genome-wide significance in pathway 1 used to create Table 3.

See Supplementary File Table_S04.xlsx

**Supplementary Table 5.** Supplemental information for Table 4. (A) Linkage disequilibrium (LD, *r^2^*) for SNPs from the pleiotropic meta-analysis in pathway 2 reported in Table 4, which are within ±1Mb flanking region. LD was evaluated in ARIC with cut off *r^2^*=0.01. (B) The results from three pleiotropic meta-analyses for SNPs attained genome-wide significance in pathway 2 used to create Table 4.

See Supplementary File Table_S05.xlsx

**Supplementary Table 6.** Pleiotropic SNPs attained genome-wide significance (*p*<5×10^‑8^) in Major Histocompatibility Complex in pathway 1b. (A) Linkage disequilibrium (LD, *r^2^*) for pleiotropic SNPs from the MHC region. LD for most SNPs was evaluated in ARIC. For rs2523567 we used FHS. Cut off was *r^2^*=0.01. (B) Summary statistics for SNPs from the MHC region attained genome-wide significance in pleiotropic meta-analysis. (C) The results from the fixed-effect meta-analysis of the associations of the MHC SNPs with each of 24 phenotypes used to create Supplementary Table 6B. (D) The results from the Fisher meta-analysis of the associations of the MHC SNPs with each of 24 phenotypes used to create Supplementary Table 6B. (E) The results from four pleiotropic meta-analyses for the MHC SNPs with each of 24 phenotypes used to create Supplementary Table 6B.

See Supplementary File Table_S06.xlsx

**Supplementary Table 7.** The results of pleiotropic meta-analyses for SNPs reported in Table 4 in each cohort separately (pathway 2a) and after Fisher meta-analysis across cohorts (pathway 2b). Meta *p-*values are provided for all three pleiotropic meta-tests (FpFc, OpFc, and ObFc) for a given domain (Fig. 1D). Cohort-specific *p-*values are given for the pleiotropic meta-analysis with the smallest *p-*value.

See Supplementary File Table_S07.xlsx

**Supplementary Table 8.** Pleiotropic meta-statistics for the antagonistic heterogeneity reported in Table 4 in each cohort separately (pathway 2a) and combined using three meta-tests, FpFc, OpFc, and ObFc in pathway 2b (Fig. 1).

See Supplementary File Table_S08.xlsx

**Supplementary Table 9.** Top 41 ingenuity canonical pathways. Canonical pathways identified using the Ingenuity Pathway Analysis software are enriched for genes at logP (=–log10(P)) ≥ 4 in at least one of four gene sets for: (i) pleiotropic SNPs from Tables 3 and 4 separately (pw1 and pw2, respectively) and combined (pw1&2) and (ii) albumin in urine (AlbU) SNPs from Table 2.

See Supplementary File Table_S09.xlsx

**Supplementary Table 10.** Top 17 Ingenuity canonical pathways for the conventional and unconventional sets.

See Supplementary File Table_S10.xlsx

**Supplementary Table S11**. Top 43 terms from Ingenuity tox list for conventional and unconventional sets.

See Supplementary File Table_S11.xlsx

**Supplementary Table 12.** Top GO terms for biological processes enriched in DAVID.

See Supplementary File Table_S12.xlsx
